# Volumetric Shaping of Nanoparticle-DNA Crystals by Light-Induced Milling

**DOI:** 10.1021/acs.nanolett.5c02830

**Published:** 2025-08-12

**Authors:** Julia M. Chmielewska, Daniel C. Redeker, Piotr Szustakiewicz, Zohar Arnon, Filip Powala, Bohdan Paterczyk, Aaron Michelson, Oleg Gang, Pawel W. Majewski

**Affiliations:** † Faculty of Chemistry, 49605 University of Warsaw, Warsaw 02-089, Poland; ‡ Department of Chemical Engineering, 5798 Columbia University, New York, New York 10027, United States; § Faculty of Biology, University of Warsaw, Warsaw 02-096, Poland; ∥ Department of Applied Physics and Applied Mathematics, 5798 Columbia University, New York, New York 10027, United States; ⊥ Center for Functional Nanomaterials, 8099 Brookhaven National Laboratory, Upton, New York 11973, United States; # Center for Nanomedicine, Institute for Basic Science (IBS), Seoul 03722, Republic of Korea

**Keywords:** DNA-programmable self-assembly, DNA-nanoparticle crystals, light-guided self-assembly, laser processing, plasmonic heating

## Abstract

DNA-programmable self-assembly enables the formation of nanoparticle crystals with controlled lattice symmetry. While this approach offers the formation of complexly ordered nanostructures for optical, mechanical, and biological applications, a mesoscale control over such nanomaterials is limited. Directing the material formation process through the assembly pathway or external fields allows for modulating crystal morphology, but achieving arbitrary morphology remains challenging. Here, we present a photothermal method for shaping 3D DNA-programmable crystals of gold nanoparticles. Through local heating of nanoparticles due to plasmonic light absorption, we induce targeted volumetric dissolution of specifically defined crystal areas with micron-scale accuracy. This technique effectively prescribes crystal shaping and creates arbitrarily shaped voids within crystals. We further investigate both computationally and experimentally the key factors governing volumetric material subtraction. The developed automated light-milling platform enables the fabrication of nanomaterials exhibiting both DNA-programmable nanoscale order and custom-designed mesoscale architecture.

## Graphical abstract

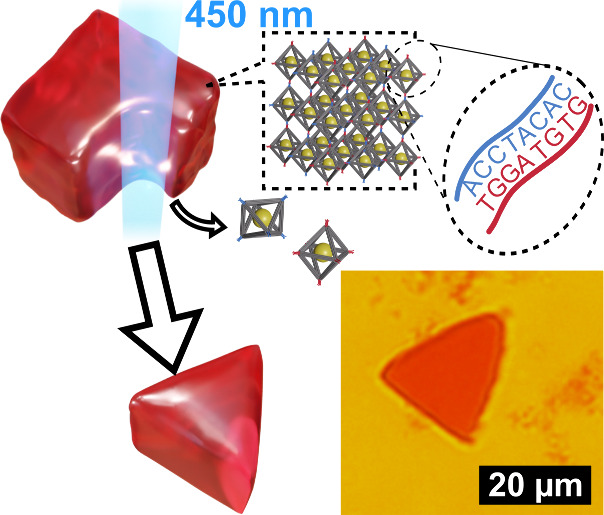

Bottom-up fabrication of nanomaterials enables the incorporation of nanoscale components, including diverse types of nanoparticles, proteins, and macromolecular complexes, into larger-scale three-dimensional (3D) organizations, which offer access to novel optical, mechanical, and chemical material properties.^1–7^ Much effort was dedicated to understanding how entropic and enthalpic factors drive the nanoscale structuring in polymeric and nanoparticle assemblies.^8–10^ Among self-assembly strategies, DNA-based methodology offers encoding of addressable bindings of nanoscale components using Watson–Crick base pair interactions.^11,12^ Structural control over the DNA nanostructures can be achieved through the formation of DNA tiles^13–15^ and origami^16,17^ that provide directional interactions.^18,19^ Together, these capabilities offer a comprehensive approach to creating DNA-programmable materials with complex internal organizations, where nanocomponents form superlattices with crystallographic symmetries determined by isotropic and anisotropic interactions, and entropic effects.^20–23^ One powerful realization of this strategy uses DNA origami polyhedral frames assembled into ordered frameworks. Frames can be loaded with nanoparticles and proteins using DNA-encoded interactions, allowing for the assembly of various nanocomponents and significantly decoupling the nanomaterial characteristics (surface functionalization, shape, polydispersity, etc.) from the self-assembly process. Different types of periodic nanostructures were demonstrated using this strategy,^20–24^ and recently developed inverse design strategies opened the possibility for creating fully programmable 3D nanomaterials.^25–27^

However, leveraging these 3D DNA-programmable nanomaterials for potential functional applications requires (i) establishing methods to make them environmentally robust, (ii) incorporating inorganic materials, and (iii) controlling the morphology of formed materials and modulation of their structure. With respect to goals (i) and (ii), the silication of 3D DNA frameworks and their templating by diverse inorganic materials, from metals to semiconductors, was demonstrated.^28,29^ This approach enhances assemblies’ thermal and mechanical resistance, maintains structural integrity outside a liquid environment, and introduces new properties based on the inorganic materials used. Regarding goal (iii), several strategies have been investigated for controlling and modulating the morphologies of assemblies using light,^30–34^ enzymatic,^35^ or chemical^36^ stimulation. In this respect, light-induced plasmonic heating offers microscale control over material removal;^31^ however, using it for shaping 3D DNA-based crystalline materials is challenging. An ability to control the morphology of DNA-based crystals was explored using interaction control of directional DNA bonds through varying axial growth rates.^37^ Patterned surfaces were used to grow crystals from DNA-coated nanoparticles in defined surface areas.^38,39^ Recently, acoustic forces were demonstrated to control the macroscale morphology of DNA framework assemblies.^40^ Despite these advances, there is no methodology to shape DNA-based crystals into predefined arbitrary forms, nor to remove material within their internal volume. Such capabilities would enable mesoscale structural control of DNA-programmable materials for emerging applications and devices. Combining nanoscale programmability with mesoscale and macroscale control is needed for a full-cycle DNA-based fabrication platform. For that reason, we utilized a light-driven approach to pattern DNA superlattices in situ with remarkable spatial and temporal control.

Light provides powerful spatiotemporal material control.^41^ Particularly, light-induced heating effects have been broadly used for manipulating soft materials at small scales.^42–45^ For example, local heating can generate strongly nonequilibrium conditions that either accelerate crystal annealing, control local optical properties of liquid crystals^46^ or guide diblock polymer structures in phases difficult to realize for equilibrium assembly.^47,48^ For DNA-functionalized gold nanoparticles, a number of studies explored how light absorption due to plasmonic effects heats the surroundings^49,50^ and melts nearby DNA duplexes, causing structural changes.^51^ Further work has demonstrated spatial control over AuNP-DNA^30^ or gold nanorod-DNA^31^ thin films through plasmonic heating, however these studies are limited to 2D assemblies. It is highly beneficial to employ light-induced heating as a local ″machining″ tool to carve crystalline 3D DNA-programmable materials into desired shapes while in solution.

The extreme heating effect produced by a laser is commonly used for shaping solid inorganic materials via ablation.^52^ These processes strongly depend on laser-material interactions and heat distribution. This approach inspired us to apply light as a source of heat for plasmonic nanoparticles due to their wavelength-specific light absorption. Local heating near particles results in DNA melting at modest temperatures in aqueous solution and dissolution of the DNA framework due to the dehybridization of bonds between nanoparticles and DNA frames, neighboring frames, and frame-internal DNA motifs. Thus, laser illumination within the plasmonic regime acts as a “light milling” and removes gold nanoparticles (AuNPs) and DNA origami frames from DNA crystals. Here, we show that the light-milling process, using a micron-sized laser beam, enables mesoscale shaping of 3D DNA-AuNP crystals, either by carving them into defined planar geometries or forming arbitrarily shaped through-voids. Our study reveals the relationship between the optical properties of AuNP-DNA superlattices and the requirement for laser illumination for effective material “machining” in solution using modeling and experiments.

The proposed light-milling strategy relies on local heating by plasmonic nanoparticles: light absorbed by assembled AuNPs raises the temperature in their vicinity.^53^ For a beam-defined illumination volume, this heat creates a zone where temperatures surpass the melting point of the DNA duplexes, forming interframe bonds, anchoring AuNPs to frames, and forming the frames themselves. At steady state, internal temperature gradients spread through the DNA superlattice; wherever melting thresholds are exceeded, material is removed from the crystal. We propose that by matching beam size, power, and dwell time with the superlattice’s thermal properties, laser milling can produce features on the order of the beam diameter. Because beam widths are tunable to microns, this strategy promises precise, controllable, on-demand mesoscale shaping of DNA crystals.

First, we briefly outline the assembly of AuNP-DNA crystals built from DNA-origami polyhedral frames. A library of staple strands and an M13 scaffold fold into 3-D octahedral frames^20,24,54^ able to internally sequester DNA-functionalized 10 nm AuNP “cargo” (Figure 1a). Adjacent frames link through vertex-to-vertex bonds supplied by complementary single-stranded DNA (so-called sticky ends), producing a simple-cubic DNA-origami framework lattice (Figure 1b). For the crystals used here, octahedra and nanoparticles were mixed, heated to 50 °C, then slowly cooled and annealed^20^ (see Supporting Note 1). During cooling, the internal oligonucleotides capture AuNPs bearing matching DNA strands, while the outer strands drive frame–frame fusion. The product is a cube-shaped superlattice (Figure 1b) that melts between 47–50 °C depending on the DNA frame monomer and salt conectrations. Alternative structures can be obtained by altering frame geometry or vertex sequences^20,24,26,27,54^ yet their macroscopic morphology still obeys the crystal’s Wulff construction. As a proof-of-concept, we therefore use the simple cubic lattice with a cube Wulff shape, giving an unambiguous starting point for visualizing the reshaping process (Figure 1c,d). But, as shown below, laser milling can be applied to different types of AuNP-DNA crystals with the adjustment of beam characteristics.

**Figure 1:**
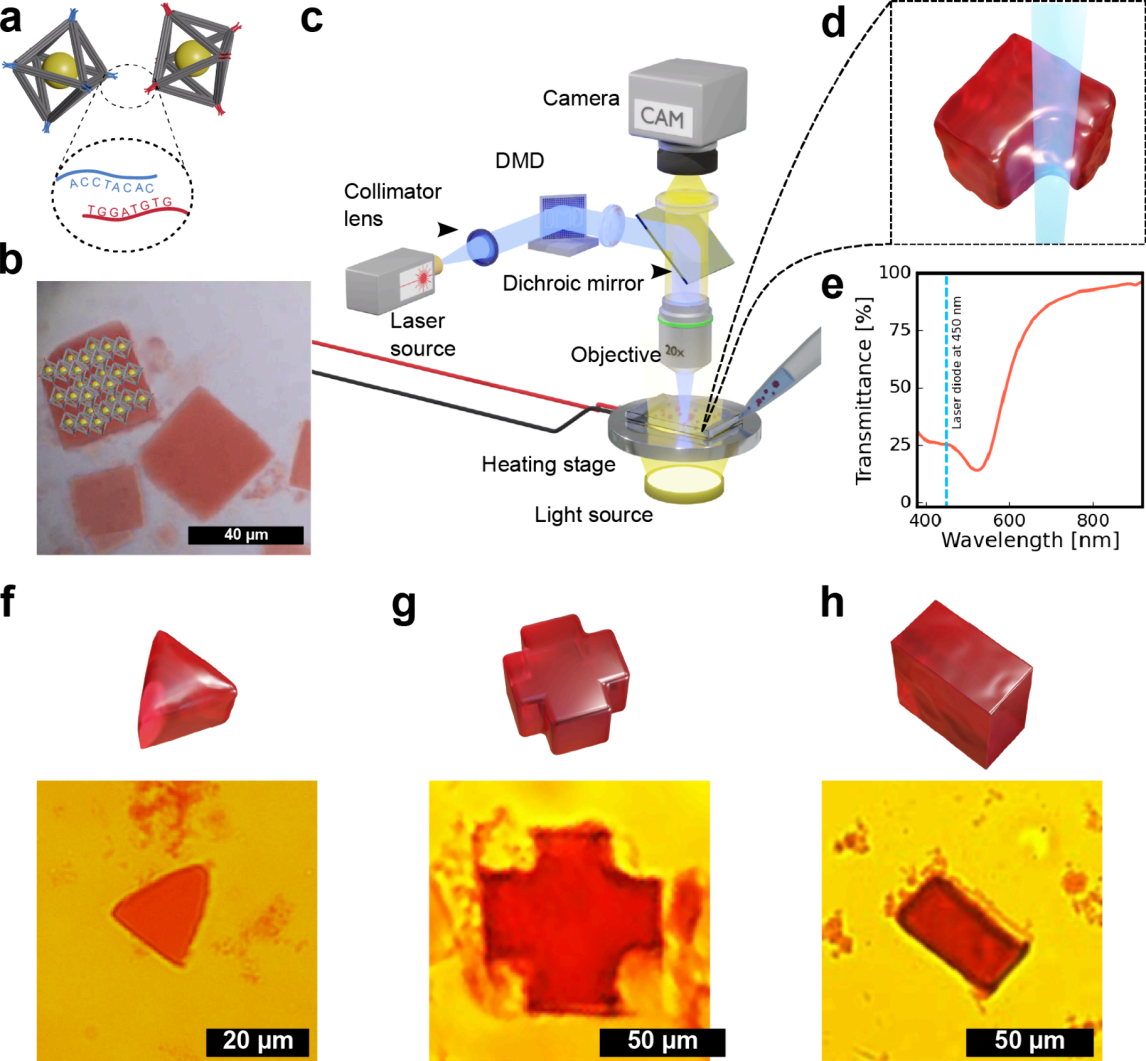
Overview of the light-milling approach for shaping DNA-based 3D nanoparticle superlattices. (a) AuNP-loaded DNA origami octahedral frames with complementary sticky end sequences at vertices (red and blue) are assembled into superlattices with simple cubic symmetry and cubic crystal morphology. (b) Optical microscope image of AuNP-DNA crystals (with structural model overlaid on the top-left crystal). (c) Schematic of the constructed laser microscope system along with (d) graphical representation of the laser sculpting process. (e) UV–vis transmission spectra of a single AuNP-DNA crystal with the laser wavelength marked by a vertical dotted line. (f)–(h) graphical models and optical images of AuNP-DNA crystals sculpted into triangular, cross, and rectangular shapes, see Movie S3 that shows shaping the AuNP-DNA crystals with a laser beam.

Since the DNA crystals are assembled through DNA hybridization at the vertices of neighboring frames, the duplexes can dissociate when the temperature increases, causing the frames to detach from the superlattice as monomers, effectively “melting” the crystal. At ∼55 °C origami also unfold, and at ∼64 °C AuNP bonds to the origami frame dehybridize. The equilibrium concentration of free building blocks increases with temperature until the crystals are fully melted.^55^ The lattice melting temperature, *T*_*m_lattice*_, can be experimentally established for various DNA origami concentrations; under conditions employed in this study, it was about 50 °C (see Supporting Note 1). The AuNP-DNA crystals are semitransparent to visible light and display a characteristic plasmonic absorption peak at 520 nm under spectroscopic evaluation due to the presence of AuNPs (Figure 1e). Moreover, the transmittance plateau universally observed in the 400–480 nm range for AuNPs allows for photothermal heating utilizing blue light, rather than more strongly absorbed green light, making the response of the material largely insensitive to the size and shape of NPs and the exciting radiation exact wavelength.^56^ The simple-cubic nanostructure of the DNA-AuNP assembly is confirmed by small-angle X-ray scattering (Figure S2), matching previous results.^20^

In order to control the microscale features of the superlattices, we employed a custom-built laser microscope system (LSM).^46^ The system includes an optical setup that allows simultaneous observation of the sample and illumination with arbitrary light patterns using a blue laser (450 nm, 15 W) projected by a micromirror device (Figure 1c). It enables highly localized heating of the crystals with a large degree of spatiotemporal control. The system also includes a motorized heating stage for precise sample positioning and temperature control.

To realize the concept of light-shaping AuNP-DNA crystals, we first studied the requirements for laser-induced heating within the crystal. Effective material removal from AuNP-DNA crystals requires a minimum photothermal excitation of AuNPs to reach the lattice melting point (Figure 1d,e). When melting occurs, bonds between DNA frames dehybridize, inducing the release of frames and AuNPs from the illuminated regions. We first placed the DNA crystals in the buffer solution inside a glass capillary that was placed on the LMS (see Supporting Note 1 for sample preparation methods), aiming to mill the external facets of the crystals as schematically shown in Figure 1e. We performed a series of semiautomated experiments in which selected crystals underwent *edge milling* by rastering a circular beam (10–30 μm, top-hat intensity profile 0.05–0.15 mW/μm^2^) along a programmed perimeter path (see Supporting Note 2). We observed in our initial experiments performed at room temperature, with the laser as the sole heating source (>0.5 mW/μm^2^), frequent uncontrolled thermophoretic displacement of the crystals once the beam overlaps with the crystal edge. Instead, an enhanced degree of control was achieved when the base temperature, *T*_*base*_, of the capillary was increased to ≈ 5 °C below the *T*_*m_lattice*_.

Employing this light-milling approach allowed us to mitigate crystal displacement by lowering illumination intensity, resulting in efficient shaping of the crystals (Figures 1f–h). The schematics of the desired shapes and their experimental realization are shown for crystals with arbitrarily defined triangular, cross-shaped, and rectangular morphologies. The milling process is automated by custom-written software (), which encompasses beam projection control and image analysis routines enabling crystal detection and closed-loop control of the photothermal process with a microscope camera. The scripts guide the laser path and follow the crystal orientation, similar to CNC machining. This approach exhibits how complex morphological shapes can be produced from conventional Wulff-shaped crystals and demonstrates the feasibility of spatially selective melting of the crystals for precise morphology control.

To understand the DNA-AuNP crystal response to different laser conditions (), we conducted finite-element simulations of the photothermal mechanism. We sought the laser flux and spot size that allow origami monomers to dissociate from the lattice and thus set the resolution limit for milling AuNP-DNA crystals. The model (Figures 2a) treats a 30 × 30 × 30 μm^3^ cube initially at *T*_*base*_ = 45 °C, thermally coupled to the surrounding liquid also at *T*_∞_ = *T*_*base*_. A circular, top-hat beam (0.2 mW/μm^2^), impinged on top-facet, follows Lambert–Beer attenuation within the crystal (see Supporting Notes 1–2). Figures 2b–d show temperatures for different spot sizes and beam powers. The peak temperature at the illuminated center rises linearly with incident flux (Figure 2d) and almost linearly with spot size (Figure 2c). The maximum temperature is reached ∼ 5 μm below the illuminated facet and, for a 9.1 μm beam, is ∼ 15 °C hotter than the surface (Figure 2e). Such conditions cross the *T*_*m_lattice*_ melting threshold, causing the stationary AuNP-DNA lattice to melt while monomers, lattice fragments, and AuNPs, are transported away from the illuminated region by diffusion and convective currents.

**Figure 2:**
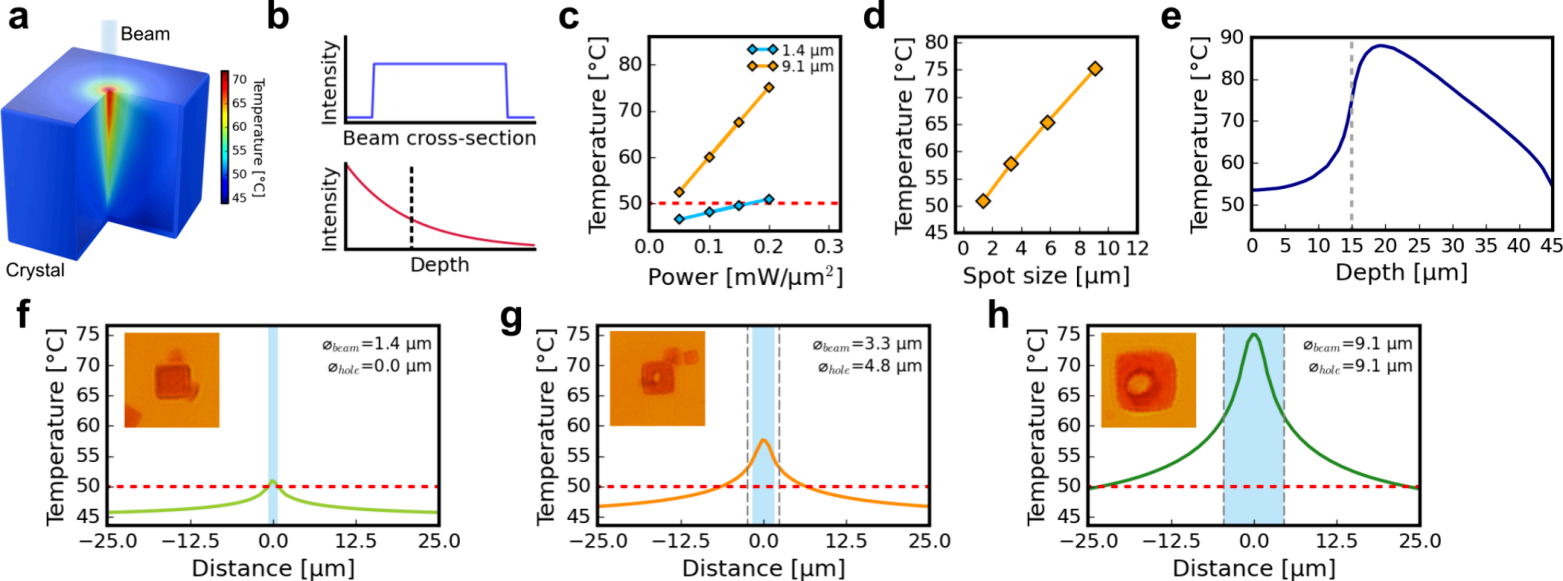
Prescribing photothermal processing of AuNP-DNA crystals by finite element simulations. (a) Simulations of steady-state temperature distribution in a cubic crystal penetrated by the beam. (b) Plots showing incident beam top-hat profile and exponentially decaying intensity in the *z*-direction due to optical absorption. (c) Maximum temperature at the top surface of the crystal as a function of laser flux for two circular 1.4 and 9.1 μm top-hat beams as a function of the laser flux. (d) Maximum temperature at the top surface of the crystal as a function of beam diameter at a constant flux of 0.2 mW/μm^2^. (e) Thermal profiles along *z*-axis positioned at the center of the beam (through crystal depth) under illumination by 9.1 μm beam at 0.2 mW/μm^2^, vertical dashed lines mark the location of the top surface of the crystal at z = 15 μm. (f–h) Simulated temperature profiles observed at the top surface of the crystal illuminated with the beams of increasing diameter (f) 1.4 μm, (g) 3.3 μm, (h) 9.1 μm at the constant flux (0.2 mW/μm ^2^) and the corresponding, microscopic images of crystals recorded after the projection of the beam onto a crystal. Blue zone marks the beam diameter, horizontal red dashed lines mark the bulk melting point of DNA lattice strands (*T*_*m_lattice*_), vertical gray dashed lines mark the experimentally observed diameter of the hole formed inside the crystal.

To determine the lattice melting threshold, we performed simulations using beams of varying diameters (1.4–9.1 μm) and illuminating the crystals with a constant intensity of 0.2 mW/μm^2^ (Figure 2f–h). We conducted corresponding experiments in which crystals were exposed to each beam for 10 s, exceeding the 100 ms required for the system to reach thermal equilibrium in simulation (Figure S9). In the experiment, beam diameters of 3.3, 5.8, and 9.1 μm produced through-holes in the crystals, with opening sizes closely matching that of the beam, while the 1.4 μm beam caused no visible changes (Figures 2f–h insets). These observations agree with the simulation results: when the temperature does not reach the melting threshold *T*_*m_lattice*_, the laser heating remains insufficient to dissociate monomers from the illuminated region.

Additionally, we assembled lattices where every other frame hosted nanoparticles and performed simulations and experiments to investigate the effect of homogeneous reduction of particle density on the photothermal effect. According to the Beer–Lambert law, the optical absorbance in these half-filled crystals should be half that of the fully filled lattice, as supported by micro UV–vis measurements showing the absorbance at 450 nm is approximately 0.25 for a 20 μm half-filled crystal (). Because power dissipation in the crystal is proportional to optical absorption (rather than absorbance), the photothermal effect for half-filled lattices is about 65% of that in a fully filled system, as evidenced by the corresponding temperature rise under identical illumination (Figure S11). The half-filled lattices are then easily experimentally processed by the LMS by increasing the laser flux to compensate for the reduced absorption.

We note that the observed time scales in laser-processing of AuNP-DNA crystals are governed by relatively slow lattice-melting kinetics rather than heat transfer. Experimentally, hole formation occurs 2–5 s after illumination begins, representing a period 2 orders of magnitude longer than required to reach a steady-state temperature distribution (Supplementary Movie S1 and Figure S9). This indicates that the lattice-melting kinetics and mass transfer ultimately dictate how quickly AuNP-DNA crystals can be laser-processed.

We next applied laser illumination to drill multiple holes in a single crystal (Figure 3a). The insights gained into the discussed thermal effects enabled us to develop automated drilling experiments. Using a 3.3 μm circular beam at an intensity of 0.2 mW/μm^2^, the drilling was automated via algorithms that identify each crystal’s position and orientation in the microscope field-of-view and align the projected hole centers on the crystal (see Supporting Note 2). Subsequently, by employing five-second beam dwells per hole, crystals featuring one to six holes arranged in a “dice” pattern were produced. Confocal and bright-field images of these crystals are shown in Figures 3b and 3c.

**Figure 3:**
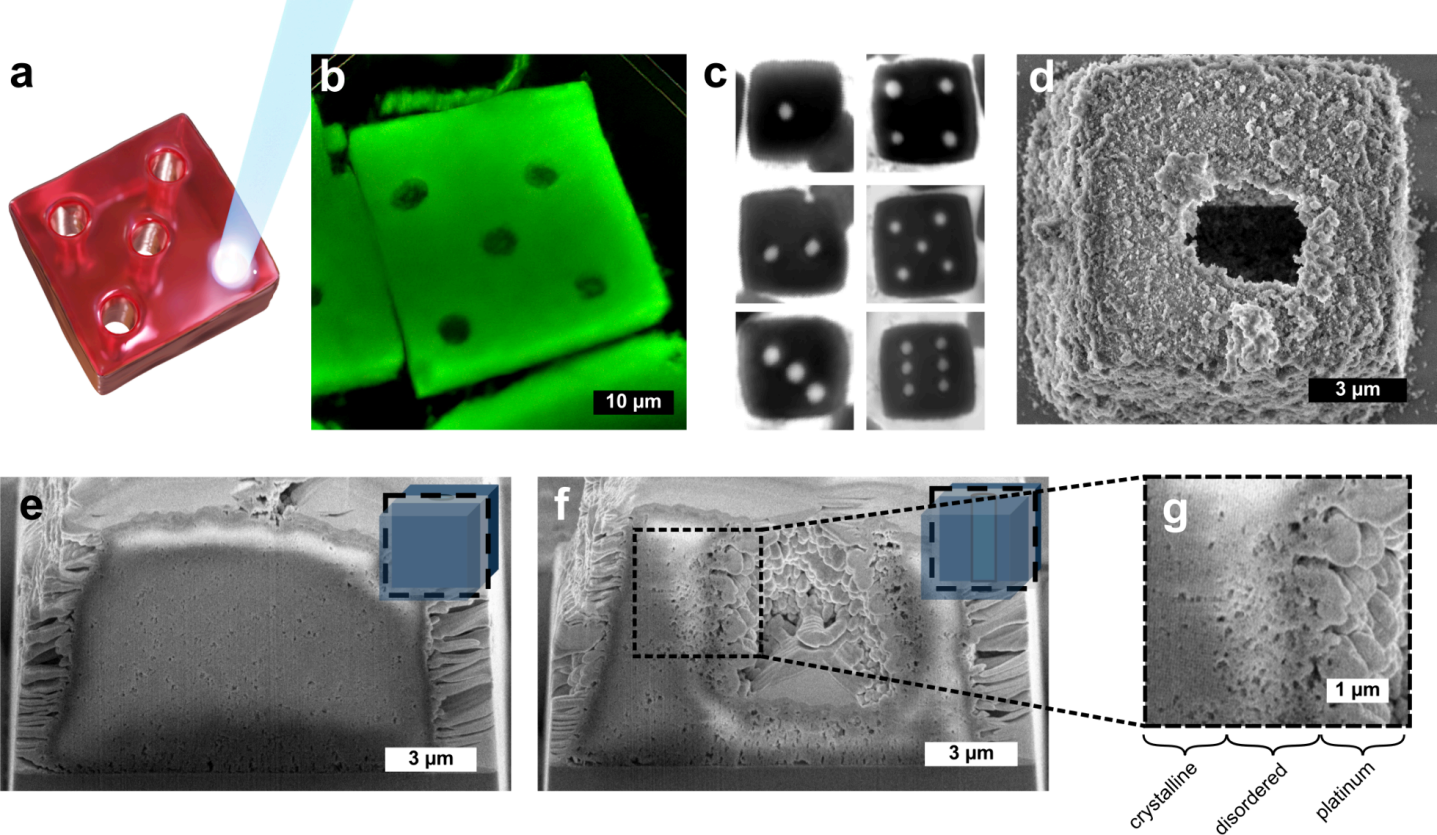
Laser drilling in AuNP-DNA crystals. (a) Light-drilling schematics, (b) and (c) dice set created by patterning a series of through-hole patterns imaged in confocal and bright field optical microscope, see Movies S1 and S2 obtained during the drilling process. (d) SEM image of laser-drilled crystal after fixation by silica coating.^29^ (e–g) FIB-SEM tomography performed on a silica-coated AuNP-DNA crystal in the plane parallel to the axis of the laser-drilled hole, see Movie S4 showing the corresponding tomographic reconstruction of drilled crystal. The light drilling in crystals was performed with a 3.3 μm circular beam with an intensity of 0.2 mW/μm^2^.

To characterize the milled regions at the nanoscale, we examined the processed crystals with FIB-SEM tomography. Silica-coated crystals^28^ were overlaid with a protective platinum layer and sectioned in 15 nm steps; each slice was imaged and assembled into a 3-D tomogram. The pre-FIB image (Figure 3d) shows the undeformed simple-cubic lattice (Figure 3e) outside the milled zone. The central slice (Figure 3f) reveals a 5.6 μm-wide, 6.1 μm-deep cylindrical void; the magnified view (Figure 3g) displays Pt infiltration and a disordered annulus, indicating partial melting at the periphery. Notably, the hole stops ∼1.7 μm short of the opposite face, indicating the beam did not fully remove the frames. To understand the incomplete breakthrough, we ran time-dependent photothermal simulations (Figure S10). As drilling proceeds, the illuminated slab thins, lowering optical absorption and, consequently, local heating. Incrementally increasing laser power can compensate for this effect. Two further limits emerge: released origami-AuNP monomers may clog the cavity, and the 50× objective’s depth-of-field causes progressive defocus, reducing energy delivery at depth. Together, these effects cap hole length as we observe experimentally; yet, power ramping, crystal reorientation, or *z*-axis translation should enable through-cuts and more elaborate three-dimensional geometries in future experiments.

Laser milling can be accelerated with multiple beams. We mapped the minimum center-to-center spacing that still yields distinct holes (Supporting Note 3). With a 3.3 μm top-hat beam at 0.2 mW/μm^2^, two holes separate cleanly at 14 μm; reducing the gap to 10 μm merges thermal fields and fuses the openings (Figure S12). Building on this insight, we created a ″laser-milling″ routine that controls multiple beams (see Movie S2) and steers the beam along arbitrary paths predefined in software (Figure 4a). The method offers fast, precise, highly repeatable machining of AuNP-DNA crystals. We milled Roman letters into individual cubes (Figure 4b) and assembled the set to spell Julius Caesar’s ″Alea iacta est″–″the die is cast″ (Figure 4c). We also demonstrated true 3-D patterning by coupling milling with crystal rotation: a through-hole was first drilled normal to one face, the capillary was then reoriented, and a second hole milled orthogonally into the adjacent face. Confocal imaging confirms two perpendicular channels intersecting inside the lattice (Figure 4d). These results show that multiplexed beams, algorithmic path planning, and simple sample maneuvers together turn photothermal milling into a versatile microfactory for sculpting DNA-origami superlattices, with feature resolution approaching the beam diameter and high patterning speed.

**Figure 4:**
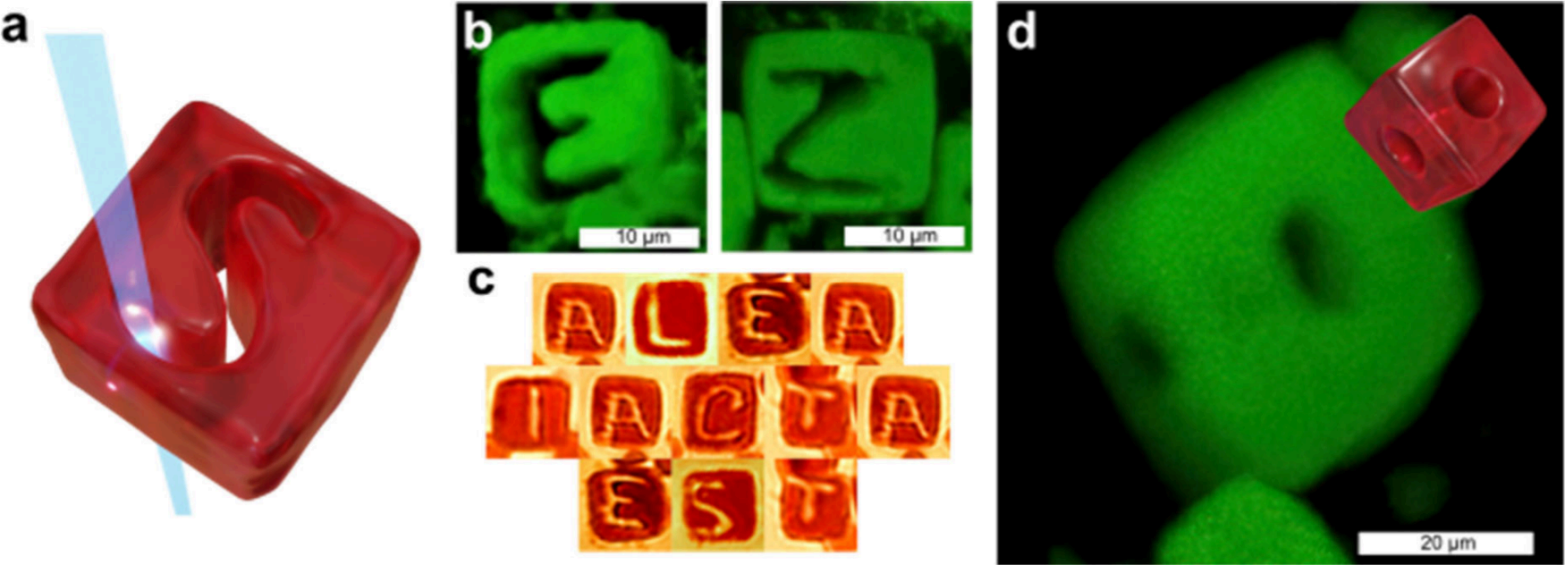
(a) Laser writing schematic. (b) Confocal images of two crystals with letters “E” and “Z” carved using the laser-milling process. (c) A Latin phrase attributed to Julius Caesar inscribed in a set of crystals. (d) Confocal image showing results of 2-step laser drilling experiment: a cubic crystal with two perpendicular holes on adjacent sides, along with a graphical model illustrating the 3D structure of the obtained crystal. The drilling was performed with 3.3 μm circular beam with an intensity of 0.2 mW/μm^2^.

In this work, the morphology of AuNP-DNA crystals was shaped volumetrically via a demonstrated ″light milling″ approach that exploits localized heating of plasmonic AuNPs embedded in DNA origami lattices. Through experimental measurements and finite-element thermal simulations, the critical milling parameters were identified. The lattice melting threshold was shown to depend on the optical absorbance of the crystals, which can be tuned by varying AuNPs loading. Once the melting threshold was surpassed, DNA frames and nanoparticles detached from the crystal, effectively carving out desirable features. Automated drilling and milling routines were developed, enabling the creation of multiple holes and user-defined patterns with micrometer precision. The tomographic characterization provided volumetric information about the void structure and nearby regions.

The successful demonstration of “light milling” for AuNP-DNA superlattices provides a versatile strategy to volumetrically process DNA-based crystalline materials with microscale precision. This technique leverages the high spatial selectivity of laser illumination and the thermally responsive nature of DNA, enabling a versatile avenue for postassembly customization of DNA nanostructures. Future setup refinement using multibeam configurations or adaptive optics, and real-time feedback could enhance throughput, resolution, and spatial control. The established approach offers a promising platform for on-demand, three-dimensional microfabrication of DNA-based nanomaterials for potential applications in photonics, sensing, and biomedicine.

## Supplementary Material











## Notes

Synthesis and characterization of AuNP-DNA crystals including SAXS and UV–vis, description of the laser microscope setup and automated milling algorithms, characterization of photothermal effects and numerical simulations, confocal microscopy with image postprocessing routines, superlattice silica coating, and additional SEM images (PDF)
Movie collected during manually controlled light-drilling of single holes in the AuNPs-DNA crystals (MP4)
Movie collected during automated light drilling of multiple holes in the AuNPs-DNA crystals (MP4)
Movie collected during automated side-milling for shaping the AuNPs-DNA crystals (MP4)
FIB-SEM tomographic reconstruction showing 3D crystal structure frame-by-frame (MP4)
