# Supplementary material for: Volumetric Shaping of Nanoparticle-DNA Crystals by Light-Induced Milling
Source: Nano Lett. 2025 Aug 12;25(34):12884–91. doi: 10.1021/acs.nanolett.5c02830 (PMC12395481; doi:10.1021/acs.nanolett.5c02830)
Supplement: Supplementary file 1 [file nl5c02830_si_001.pdf]

Volumetric Shaping of Nanoparticle-DNA Crystals by Light-Induced Milling

Supporting Information

Julia Chmielewska[1#], Daniel C. Redeker[2#], Piotr Szustakiewicz[1#], Zohar Arnon[2], Filip Powala[1], Bohdan Paterczyk[3], Aaron Michelson[5], Oleg Gang[2,4,5], Pawel W. Majewski[1]**

# Contributed equally

**Email: pmajewski@chem.uw.edu.pl; og2226@columbia.edu*

1. Faculty of Chemistry, University of Warsaw, Warsaw, 02089, Poland

2. Department of Chemical Engineering, Columbia University, New York, NY, USA

3. Faculty of Biology, University of Warsaw, Warsaw, Poland

4. Department of Applied Physics and Applied Mathematics, Columbia University, New York, NY, USA

5. Center for Functional Nanomaterials, Brookhaven National Laboratory, Upton, NY, USA

Contents

Supporting Note 1: Synthesis and characterization of AuNP-DNA crystals 3

Synthesis of DNA origami octahedra 3

Grafting gold nanoparticles with DNA 3

AuNP-DNA superlattice synthesis... 3

DNA origami staple sequences 3

Synthesis schematics for fully-filled and half-filled superlattices 10

SAXS characterization – fully-filled AuNP-DNA lattice 11

Optical characterization – fully-filled AuNP-DNA lattice 13

Optical characterization - half-filled AuNP-DNA crystals 14

Supporting Note 2: Laser microscope setup, samples' preparation, and automated milling algorithms 15

Sample preparation 15

Photothermal processing setup.. 15

Laser milling and drilling experiments 16

Supporting Note 3: Characterization of photothermal effects and numerical simulations 17

Laser flux density measurements.. 17

Numerical simulations of laser heating... 17

Temperature distribution along the optical axis of the beam 19

Time-dependent photothermal heating simulations 20

The effect of absorbing AuNPs-containing frames removal... 21

Half-filled AuNP-DNA crystals.. 22

Simultaneous drilling in two spots.. 23

Supporting Note 4: Confocal imaging and image post-processing routines 24

Confocal fluorescence microscopy 24

Supporting Note 5: Superlattice silica coating and scanning electron microscopy 25

Superlattice silica coating 25

FIB-SEM imaging.. 25

Description of supplementary video files 27

List of references 27

Supporting Note 1: Synthesis and characterization of AuNP-DNA crystals

Synthesis of DNA origami octahedra

DNA origami octahedral frames were designed and synthesized as shown previously.[1] In short, the DNA origami were formed by mixing 40 nM M13mp18 scaffold DNA (Bayou Biolabs) with a 5× excess of staple strands (IDT DNA) in 1×TAE buffer (40 mM Tris acetate, 1 mM EDTA) with 12.5 mM $MgCl_2$. This solution was heated to 90 °C to melt any preformed duplexes and slowly cooled to room temperature over 20h to fold the desired origami structure. DNA origami were washed 4–5 times in 100 kDa Amicon Centrifugal Filters to remove excess staples, and the final concentration was measured with UV-Vis spectroscopy.

Grafting gold nanoparticles with DNA

DNA oligonucleotides with 3' thiol modification were purchased from IDT DNA. Thiol-modified DNA was mixed with tris(2-carboxyethyl)phosphine hydrochloride (TCEP) and incubated for 30 minutes. The TCEP-DNA mixture was then mixed with 10 nm gold nanoparticles with a 180:1 excess of DNA to particles and placed on an orbital shaker and incubated for 1 hour. Afterwards, phosphate buffer was slowly added to reach a 10 mM phosphate concentration. In order to further stabilize the negatively charged DNA strands on the particle surface, NaCl was slowly added in 5 steps with 30-minute incubation periods to a final concentration of 300 mM. Particles were washed in 50 kDa Amicon filters after incubating overnight to remove excess DNA, and the final concentration was measured with UV-Vis spectroscopy.

AuNP-DNA superlattice synthesis

DNA origami superlattices were synthesized by mixing DNA origami octahedra with complementary sticky ends placed on the vertices. Typically, a mixture of 20 nM of each origami was mixed with DNA grafted AuNPs with a 2× excess and heated to 50 °C, slowly cooled to 35 °C at a rate of 0.2 °C per hour, then cooled more rapidly to room temp over several hours. For Half-Filled superlattices, one of the origami used does not contain particle binding internal sticky ends, and the 2x excess of DNA grafted AuNPs is adjusted accordingly.

DNA origami staple sequences

Table S1. *Non particle binding internal staple sequences for octahedron origami.*

Sequence Name	Sequence
Octa_Staple1	GCCTTGAATCTTTTCCGGAAC-CGCCTCCCAGAGCCCAGAGCCGCCGCCAGCATT
Octa_Staple2	ATAAGGCGCCAAAAGTTGAGATTTAGGATAACGGACCAGTCA
Octa_Staple3	GATGGTTGGGAAGAAAAATCCACCAGAAATAATTGGGCTTGA
Octa_Staple4	GGACGTTTAATTTCGACGAGAAACACCACCACTAATGCAGAT
Octa_Staple5	ATTTTAAGAACTGGCTTGAATTATCAGTGA
Octa_Staple6	TCAGAACCGCCACCCTCTCAGAGTATTAGC
Octa_Staple7	TCAGAGCGCCACCACATAATCAAAATCAGAACGAGTAGTATG
Octa_Staple8	AACCAGACGCTACGTTAATAAAACGAACATACCACATTCAGG
Octa_Staple9	GTTTGCCTATTCACAGGCAGGTCAGACGCCACCACACCACCC

Octa_Staple10	ACATAACTTGCCCTAACTTTAATCATT-GCATTATAACAACATTATTACAGGTAG
Octa_Staple11	CATGTCACAAACGGCATTAAATGTGAGCAATTCGCGTTAAAT
Octa_Staple12	AAGATTGTTTTTAACCAAGAAACCATCGACCCAAAAACAGG
Octa_Staple13	CAGCTCATATAAGCGTACCCCGGTTGATGTGTCGGATTCTCC
Octa_Staple14	GTTAAAATTCGCATTATAAACGTAAACTAG
Octa_Staple15	AGCACCATTACCATTACAGCAAATGACGGA
Octa_Staple16	GTCACCAGAGCCATGGTGAATTATCACCAATCAGAAAAGCCT
Octa_Staple17	CCGACTTATTAGGAACGCCATCAAAAATGAGTAACAACCCCA
Octa_Staple18	AATTATTGTTTTCATGCCTTTAGCGTCAGATAGCACGGAAAC
Octa_Staple19	CTTCGCTGGGCGCAGACGACAGTATCGGGGCAC-CGTCGCCATTCAGGCTGCGCA
Octa_Staple20	ACAAAGAAATTTAGGTAGGGCTTAATTGTATACAACGGAATC
Octa_Staple21	TGACCTACTAGAAAAAGCCCCAGGCAAAGCAATTTCATCTTC
Octa_Staple22	ATAATTAAATTTAAAAAACTTTTTCAAACTTTTAACAACGCC
Octa_Staple23	AGGCGTTAAATAAGAAGACCGTGTCGCAAG
Octa_Staple24	TGCCGGAAGGGGACTCGTAACCGTGCATTATATTTTAGTTCT
Octa_Staple25	CTCCAGCCAGCTTTCCCCTCAGGACGTTGG
Octa_Staple26	CAGTTTGAATGTTTAGTATCATATGCGTAGAATCGCCATAGC
Octa_Staple27	TGTAGATATTACGCGGCGATCGGTGCGGGCGCCATCTTCTGG
Octa_Staple28	AACATGTACGCGAGTGGTTTGAAATACCTAAACACATTCTTACCAG-TATAAAGC
Octa_Staple29	CGCTGGTGCTTTCCTGAATCGGCCAACGAGGGTGGTGATTGCCCTTCAC-CGCCT
Octa_Staple30	AACAAAAATAACTAGGTCTGAGAGACTACGCTGAGTTTCCCT
Octa_Staple31	AACAGTACTTGAAAACATATGAGACGGGTCTTTTTTAATGGA
Octa_Staple32	TAGAATCCATAAATCATTTAACAATTTCTCCCGGCTTAGGTT
Octa_Staple33	GTAAATCGTCGCTATTGAATAACTCAAGAA
Octa_Staple34	TTGCGTATTGGGCGCCCGCGGGGTGCGCTC
Octa_Staple35	TTTCACCGCATTAAAGTCGGGAAACCTGATTTGAATTACCCA
Octa_Staple36	GCCAGCTAGGCGATAGCTTAGATTAAGACCTTTTTAACCTGT
Octa_Staple37	ACTGCCCTTGCCCCGTTGCAGCAAGCGGCAACAGCTTTTTCT
Octa_Staple38	GGGTTATTTAATTACAATATATGTGAGTAATTAATAAGAGTCAA-TAGTGAATTT
Octa_Staple39	TCCAAATCTTCTGAATTATTTGCACGTAGGTTTAACGCTAAC-GAGCGTCTTTCC
Octa_Staple40	CAGATATTACCTGAATACCAAGTTACAATCGGGAGCTATTTT
Octa_Staple41	ACGCGAGGCTACAACAGTACCTTTTACAAATCGCGCAGAGAA
Octa_Staple42	GCACCCAGCGTTTTTTATCCGGTATTCTAGGCGAATTATTCA
Octa_Staple43	AAGCCTTAAATCAAGACTTGCGGAGCAAAT
Octa_Staple44	ATTGCGTAGATTTTCAAAACAGATTGTTTG
Octa_Staple45	TGAATATTATCAAAATAATGGAAGGGTTAATATTTATCCCAA
Octa_Staple46	CCTACCAACAGTAATTTTATCCTGAATCAAACAGCCATATGA
Octa_Staple47	GATTATAAAGAAACGCCAGTTACAAAATTTACCAACGTCAGA
Octa_Staple48	TTTCAATAGAAGGCAGCGAACCTCCCGATTAGTTGAAACAATAACGGAT-TCGCC

Octa_Staple49	GATATTCTAAATTGAGCCGGAACGAGGCCCAACTTGGCG-CATAGGCTGGCTGAC
Octa_Staple50	GGTTGATTTTCCAGCAGACAGCCCTCATTCGTCACGGGATAG
Octa_Staple51	AGTACCGAATAGGAACCCAAACGGTGTAACCTCAGGAGGTTT
Octa_Staple52	CAAGCCCCCACCCTTAGCCCGGAATAGGACGATCTAAAGTTT
Octa_Staple53	CAGAGCCACCACCCTCTCAGAACTCGAGAG
Octa_Staple54	AAGGGAACCGAACTGAGCAGACGGTATCAT
Octa_Staple55	GGACAGAGTTACTTTGTCGAAATCCGCGTGTATCACCGTACG
Octa_Staple56	GCTCCATTGTGTACCGTAACACTGAGTTAGTTAGCGTAACCT
Octa_Staple57	CGCCTGAATTACCCTAATCTTGACAAGACAGACCATGAAAGA
Octa_Staple58	TGTCGTCATAAGTACAGAACCGCCACCCATTTTCACAGTACAAAC-TACAACGCC
Octa_Staple59	ATGACCACTCGTTTGGCTTTTGCAAAAGTTAGACTATATTCATT-GAATCCCCCT
Octa_Staple60	GTAATACGCAAACATGAGAGATCTACAACTAGCTGAGGCCGG
Octa_Staple61	AGAACCCCAAATCACCATCTGCGGAATCGAATAAAAATTTTT
Octa_Staple62	AGACAGTTCATATAGGAGAAGCCTTTATAACATTGCCTGAGA
Octa_Staple63	GTAAAGATTCAAAAGGCCTGAGTTGACCCT
Octa_Staple64	GGTAATAGTAAAATGTAAGTTTTACACTAT
Octa_Staple65	GTCCAATAGCGAGAACCAGACGACGATATTCAACGCAAGGGA
Octa_Staple66	CCAAAATACAATATGATATTCAACCGTTAGGCTATCAGGTAA
Octa_Staple67	CATAACCTAAATCAACAGTTCAGAAAACGTCATAAGGATAGC
Octa_Staple68	GTCTGGATTTTGCGTTTTAAATGCAATGGTGAGAAATAAATTAATGCCG-GAGAG
Octa_Staple69	GGGCGACCCCAAAAGTATGTTAGCAAACTAAAAGAGTCACAATCAA-TAGAAAAT
Octa_Staple70	TATAAAGCATCGTAACCAAGTACCGCACCGGCTGTAATATCC
Octa_Staple71	CAACATGATTTACGAGCATGGAATAAGTAAGACGACAATAAA
Octa_Staple72	CATCCTATTCAGCTAAAAGGTAAAGTAAAAAGCAAGCCGTTT
Octa_Staple73	GATAAGTCCTGAACAACTGTTTAAAGAGAA
Octa_Staple74	TAAAGGTGGCAACATAGTAGAAAATAATAA
Octa_Staple75	AGACACCTTACGCAGAACTGGCATGATTTTCTGTCCAGACAA
Octa_Staple76	CTCCTTAACGTAGAAACCAATCAATAATTCATCGAGAACAGA
Octa_Staple77	CGGAATAATTCAACCCAGCGCCAAAGACTTATTTTAACGCAA
Octa_Staple78	TTATTTTTACCGACAATGCAGAAC-GCGCGAAAAATCTTTCCTTATCATTCCAAG
Octa_Staple79	CAGCCTTGGTTTTGTATTAAGAGGCTGACTGCCTATATCAGA
Octa_Staple80	GGAAGCGCCCACAAACAGTTAATGCCCCGACTCCTCAAGATA
Octa_Staple81	GAGATAACATTAGAAGAATAACATAAAAAGGAAGGATTAGGA
Octa_Staple82	GTCAGAGGGTAATTGAGAACACCAAAATAG
Octa_Staple83	AAGTTTTAACGGGGTCGGAGTGTAGAATGG
Octa_Staple84	CAGTGCCTACATGGGAATTTACCGTTCCACAAGTAAGCAGAT
Octa_Staple85	AGCGTCACGTATAAGAATTGAGTTAAGCCCTTTTTAAGAAAG
Octa_Staple86	AAAGCGCCAAAGTTTATCTTACCGAAGCCCAATAATGAGTAA
Octa_Staple87	TGCTAAACAGATGAAGAAACCACCAGAATTTAAAAAAAGGCT
Octa_Staple88	GAGAATAGAGCCTTACCGTCTATCAAATGGAGCGGAATTAGA

Octa_Staple89	CCAAAAGGAAAGGACAACAGTTTCAGCGAATCATCATATTCC
Octa_Staple90	TTCACGTTGAAAATCTTGCGAATGGGATTT
Octa_Staple91	GTCCACTATTAAAGAACCAGTTTTGGTTCC
Octa_Staple92	TCAAAGGGAGATAGCCCTTATAAATCAAGACAACAACCATCG
Octa_Staple93	ATAGCCCGCGAAAATAATTGTATCGGTTCGCCGACAATGAGT
Octa_Staple94	GAAATCGATAACCGGATACCGATAGTTGTATCAGCTCCAACG
Octa_Staple95	ATTAAGTATAAAGCGGCAAGGCAAAGAAACTAATAGGGTACC
Octa_Staple96	CACGACGAATTCGTGTGGCATCAATTCTTTAGCAAAATTACG
Octa_Staple97	CAGGTCGACTCTAGAGCAAGCTTCAAGGCG
Octa_Staple98	TAACCTGTTTAGCTATTTTCGCATTCATTC
Octa_Staple99	GAGCTCGTTGTAAACGCCAGGGTTTTCCAAAGCAATAAAGCC
Octa_Staple100	CGCGAGCTTAGTTTTTCCCAATTCTGCGCAAGTGTAAAGCCT
Octa_Staple101	AGTAGATTGAAAAGAATCATGGTCATAGCCGGAAGCATAAGT
Octa_Staple102	CATATAACTAATGAACACAACATACGAGCTGTTTCTTTGGGG
Octa_Staple103	ATGTTTTGCTTTTGATCGGAACGAGGGTACTTTTTCTTTTGATAA-GAGGTCATT
Octa_Staple104	AGAAGCAACCAAGCCAAAAGAATACACTAATGCCAAAACTCC
Octa_Staple105	GAGGAAGCAGGATTCGGGTAAAATACGTAAAACACCCCCCAG
Octa_Staple106	AACAGGTCCCGAAATTGCATCAAAAAGATCTTTGATCATCAG
Octa_Staple107	TCAAAGCGAACCAGACCGTTTTATATAGTC
Octa_Staple108	GCTTTGAGGACTAAAGAGCAACGGGGAGTT
Octa_Staple109	AAGTTTCAGACAGCCGGGATCGTCACCCTTCTGTAGCTCAAC
Octa_Staple110	CAGCGAACATTAAAAGAGAGTACCTTTACTGAATATAATGAA
Octa_Staple111	AAAGGCCAAATATGTTAGAGCTTAATTGATTGCTCCATGAGG
Octa_Staple112	CGATTATAAGCGGAGACTTCAAATATCGCGGAAGCCTACGAAGGCAC-CAACCTA

Table S2. *Particle binding internal staple sequences for octahedron origami. For particle loading origami, omit the binding sequence portion of the staples.*

**Sequence Name**	**Sequence**
Octa_InternalBinding1	GTAGCGCCATTAAATTGGGAATTAGAGCGCAAGGCGCACCG-TAATCAGTAGCGA + Binding Sequence
Octa_InternalBinding2	GTGGGAAATCATATAAATATTTAAATTGAATTTTT-GTCTGGCCTTCCTGTAGCC + Binding Sequence
Octa_InternalBinding3	AGCCGAAAGTCTCTCTTTTGATGATACAAGTGCCTTAAGAGCAA-GAAACAATGA + Binding Sequence
Octa_InternalBinding4	TTAGCGGTACAGAGCGGGAGAATTAACTGCGCTAATTTCGGAAC-CTATTATTCT + Binding Sequence
Octa_InternalBinding5	CCCACGCGCAAAATGGTTGAGTGTTGTTCGTGGACTT-GCTTTCGAGGTGAATTT + Binding Sequence
Octa_InternalBinding6	TGATTATCAACTTTACAACTAAAGGAATCCAAAAAGTTTGAG-TAACATTATCAT + Binding Sequence
Octa_InternalBinding7	GGGGTGCCAGTTGAGACCATTAGATACAATTTTCAC-TGTGTGAAATTGTTATCC + Binding Sequence
Octa_InternalBinding8	TCAGAGCTGGGTAAACGACGGCCAGTGCGATCCCCGTAGTAG-CATTAACATCCA + Binding Sequence
Binding Sequence (Octa-hedron side)	ATCCATCACTTCATACTCTACGTTGTTGTTGTTGTTGTT
Thiolated Binding Se-quence (Particle Side)	TAT GAA GTG ATG GAT GAT + Thiol Modification

__Table S3.__ Octahedron external sticky end staple sequences.

Sequence Name	Sequence
Octa_VertexA_1	AGAGCCTAATTTGATTTTTTGTTTAAATCCTGAAATAAA-GAATTTTTTTTTTTTTTTTTTTTTTT + Binding Sequence
Octa_VertexA_2	TGTAGCATTCCAACGTTAG-TAAATGAAGTGCCGCGCCACCCTTTTTTTTTTTTTTTTTTTTTT + Binding Sequence
Octa_VertexA_3	GAAACATGAAAGCTCAGTAC-CAGGCGAAAAATGCTGAACAAATTTTTTTTTTTTTTTTTTTTTT + Binding Se-quence
Octa_VertexA_4	TTTGCGGAACAATGGCAATTCATCAATCTGTATAA-TAATTTTTTTTTTTTTTTTTTTTTTTTTTT + Binding Sequence
Octa_VertexB_1	AAAGATTCATCAGGAATTAC-GAGGCATGCTCATCCTTATGCGTTTTTTTTTTTTTTTTTTTTT + Binding Se-quence
Octa_VertexB_2	CTTCATCAAGAGAAATCAACGTAACAGAGATTT-GTCAATCATTTTTTTTTTTTTTTTTTTTTTTT + Binding Sequence
Octa_VertexB_3	CAAATGCTTTAAAAAATCAGGTCTTTAAGAG-CAGCCAGAGGGTTTTTTTTTTTTTTTTTTTTT + Binding Sequence
Octa_VertexB_4	AAACGAAAGAGGGCGAAACAAAGTACTGACTA-TATTCGAGCTTTTTTTTTTTTTTTTTTTTTTT + Binding Sequence
Octa_VertexC_1	AGCTTTCATCAACGGATTGACCGTAAAATCGTATAA-TATTTTTTTTTTTTTTTTTTTTTTTTTT + Binding Sequence
Octa_VertexC_2	ACTGTTGGGAAGCAGCTGGCGAAAGGATAGGTCAAGATCG-CATTTTTTTTTTTTTTTTTTTTTTT + Binding Sequence
Octa_VertexC_3	GGTAGCTATTTTAGAGAATCGATGAAAACATTAAATGTGTAG-TTTTTTTTTTTTTTTTTTTTTT + Binding Sequence
Octa_VertexC_4	ATAAATCATACATAAATCGGTTGTACTGTGCTGG-CATGCCTGTTTTTTTTTTTTTTTTTTTTTT + Binding Sequence
Octa_VertexD_1	TGATTGCTTTGAGCAAAAGAAGATGAAATAGCAGAGGTTTT-GTTTTTTTTTTTTTTTTTTTTTTT + Binding Sequence
Octa_VertexD_2	AACGGGTATTAAGGAATCATTACCGCCAGTAATTCAACAA-TATTTTTTTTTTTTTTTTTTTTTTT + Binding Sequence
Octa_VertexD_3	CAACGCTCAACAGCAGAGGCATTTTCAATCCAATGATAAA-TATTTTTTTTTTTTTTTTTTTTTTT + Binding Sequence
Octa_VertexD_4	ATCAAAATCATATATGTAAATGCTGAACAAACACTT-GCTTCTTTTTTTTTTTTTTTTTTTTTTT + Binding Sequence
Octa_VertexE_1	GGCCCTGAGAGAAGCAGGCGAAAATCATTGCG-TAGAGGCGGTTTTTTTTTTTTTTTTTTTTTT + Binding Sequence
Octa_VertexE_2	CTTAAACAGCTTATATATTCGGTCGCTTGATGGGGAACAAGAT-TTTTTTTTTTTTTTTTTTTTT + Binding Sequence
Octa_VertexE_3	GCTCACAATTCCGTGAGCTAACTCACTG-GAAGTAATGGTCAATTTTTTTTTTTTTTTTTTTTTT + Binding Sequence

Octa_VertexE_4	TTTGCGGATGGCCAACTAAAGTACGGGCTTGCAGCTACAGAG-TTTTTTTTTTTTTTTTTTTTTT + Binding Sequence
Octa_VertexF_1	GACAGGAGGTTGAAACAAA-TAAATCCGCCCCCTCCGCCACCCTTTTTTTTTTTTTTTTTTTTT + Binding Sequence
Octa_VertexF_2	CAGAATCAAGTTTCGGCATTTTCGGTTAAATATATCACCAG-TTTTTTTTTTTTTTTTTTTTTTT + Binding Sequence
Octa_VertexF_3	TCATATGGTTTACGATTGAGGGAGGGAAACGCAATACATA-CATTTTTTTTTTTTTTTTTTTTT + Binding Sequence
Octa_VertexF_4	AATAGCAATAGCACCAGAAGGAAACCTAAAGCCACTGG-TAATTTTTTTTTTTTTTTTTTTTTTT + Binding Sequence
Binding Sequence1	ACCTACAC
Binding Sequence2	GTGTAGGT

Synthesis schematics for fully-filled and half-filled superlattices

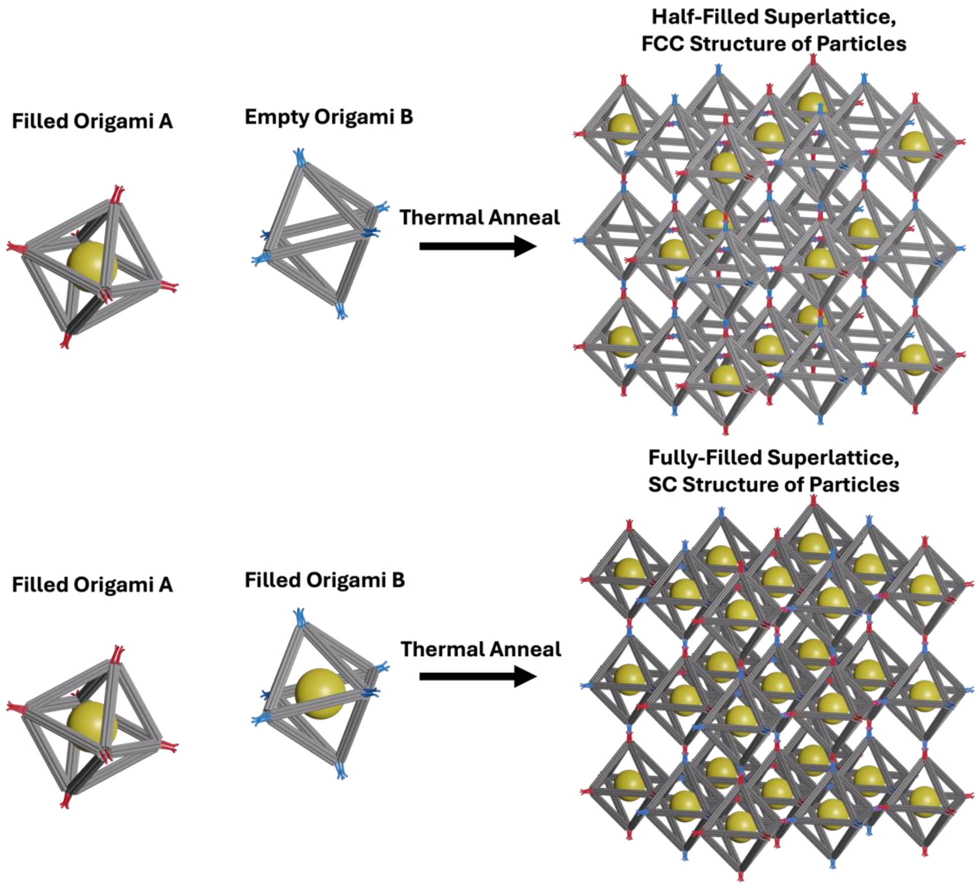

Figure S1. *Schematic showing assembly of half-filled and fully-filled DNA origami-AuNP superlattices.*

SAXS characterization – fully-filled AuNP-DNA lattice

SAXS measurements confirming the crystalline assembly of AuNP-DNA origami superlattices were performed at the Complex Materials Scattering (CMS) beamline at 11-BM and the National Synchrotron Light Source II at Brookhaven National Laboratory in Upton, NY. Samples were loaded into 1 mm diameter Boron Rich glass capillaries (Charles Supper) and allowed to settle. 2-Dimensional (2D) scattering data was measured on area detectors downstream from the sample holder. Raw 2D scattering patterns were azimuthally integrated into a one-dimensional scattering intensity curve *I(q)* as a function of the scattering wave vector q, $q = 4\frac{\pi}{\lambda}\sin\left(\frac{\theta}{2}\right)$ where λ is the wavelength of the incident x-rays and θ is the scattering angle.

Table S4. *X-ray beam characteristics and beamline setup details.*

Beamline	11-BM CMS
Photon Energy (keV)	13.5
Horizontal × Vertical Beam Size (μm × μm)	200 × 200
Flux (photons/sec)	10^{11}
Detector Distance (m)	5.05
Detector Manufacturer	Dectris
Detector Model	Pilatus 1M
Detector Pixel Size (μm × μm)	172 × 172

The structure factor was obtained by dividing *I(q)* by the corresponding particle form factor *P(q)*. In this work, we implement modelling of the presented analysis using the ScatterSim software package,[2] a Python package that implements a scattering formalism for superlattices. This formalism can generically model the scattering from arbitrary anisotropic nano-objects within the unit cell of a regular superlattice. We used this library to perform the modelling for the simple cubic crystal, with the code being available for download on Git Hub.[2]

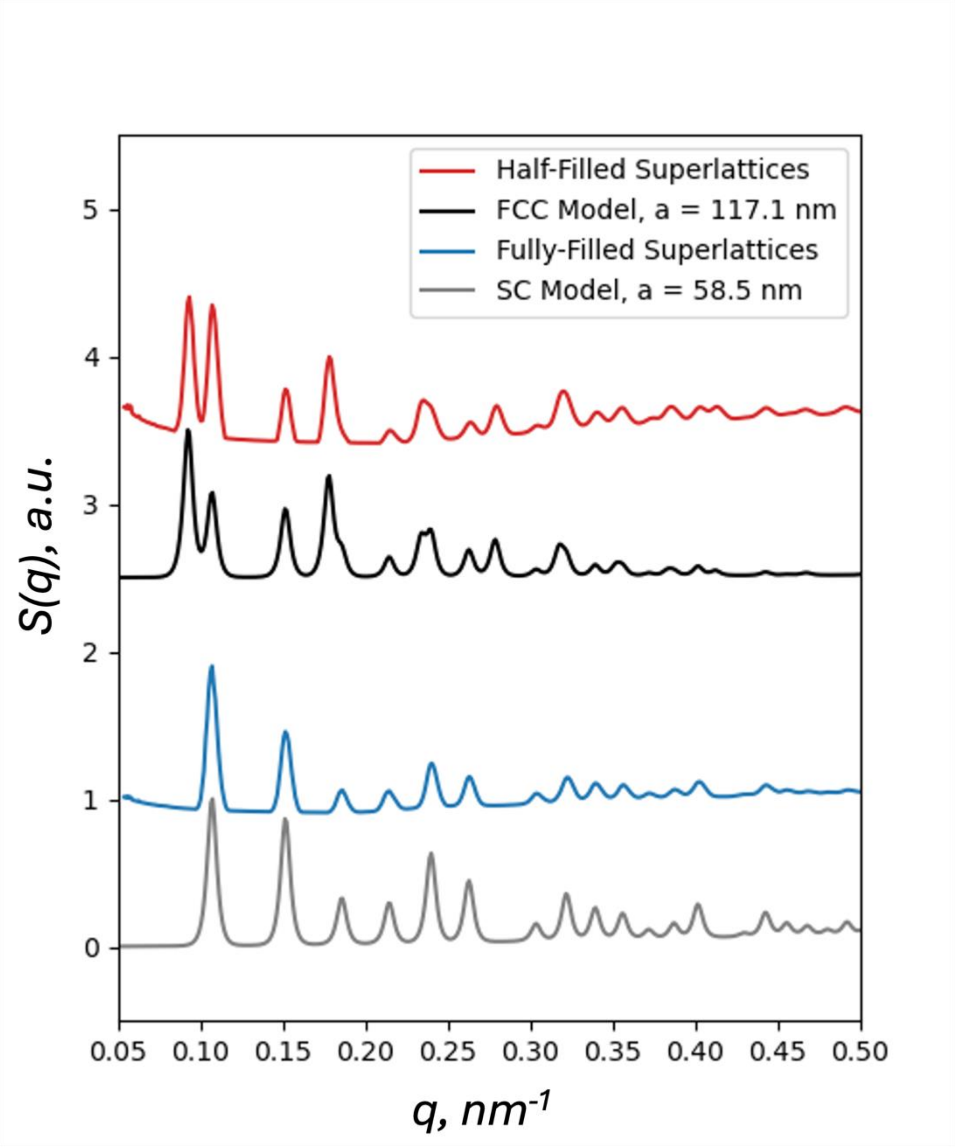

Figure S2. *Structure factors from small-angle x-ray scattering characterization of fully-filled superlattices (blue) with a simple cubic crystal model (grey) and half-filled simple-cubic superlattices (red) that form a face-centered cubic crystal (SAXS model shown with black line).*

Optical characterization – fully-filled AuNP-DNA lattice

Micro UV-VIS spectroscopic measurements were performed using an upright white-field microscope (Zeiss, ImagerA2m) equipped with a selective area aperture located at the intermediate imaging plane between the objective (Zeiss, EC Epiplan-Neofluar, 50×, NA 0.55) and the imaging camera. The minimum size of the circular aperture corresponds to 20 µm in the object plane. The crystals confined in a glass capillary were illuminated by a narrow cone of white light (NA ≈ 0.2) projected by a microscope illumination optics set up in the Koehler configuration. The white light source was a tungsten-halogen bulb with an IR filter removed. A 400 µm core multimode fiber optics equipped with an achromatic collector lens mounted in one of the microscope camera ports was used to collect the transmitted light and to relay it to an optical spectrometer. The optical spectra were recorded in the 380–1000 nm spectral range after positioning the centers of individual crystals confined in the glass capillary over the selective area aperture; a reference background spectrum was acquired over a region containing the crystal growth buffer.

The size of AuNP-DNA crystals determines how much laser light they absorb. To examine this effect, we measured crystal dimensions from optical images (Figure S3a) and recorded individual crystal absorbance at 450 nm (Figure S3b). The data show a linear correlation between crystal size and absorbance, following the Beer-Lambert law with a characteristic intensity decay length of approximately 23 µm. In other words, about 63% of incident photons are absorbed within this depth of the material. These findings explain the strong photothermal response observed in AuNP-DNA crystals.

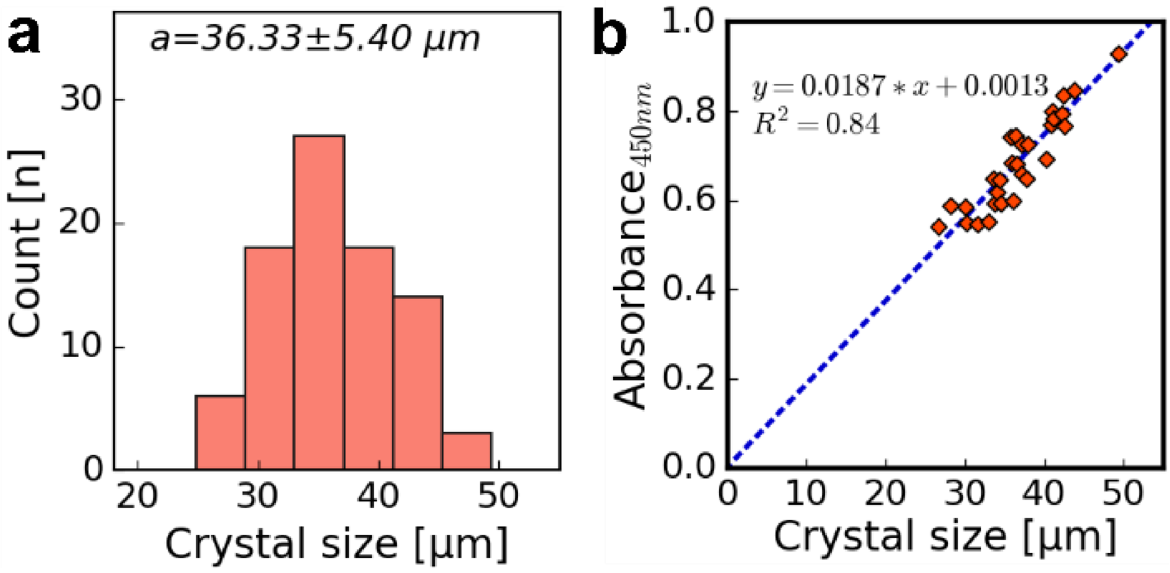

Figure S3. *Micro UV-Vis characterization of fully-filled AuNP-DNA crystals. (a) The histogram depicts the lateral size distribution of AuNP-DNA crystals. (b) The correlation between the lateral crystal size and absorbance at 450 nm.*

Optical characterization - half-filled AuNP-DNA crystals

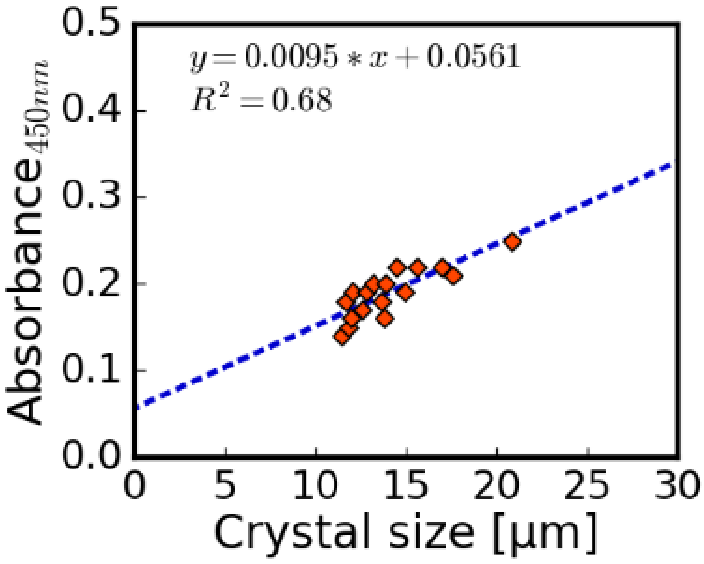

Figure S4. *Micro UV-Vis characterization of half-filled AuNP-DNA crystals. The correlation between the lateral crystal size and absorbance at 450 nm.*

Supporting Note 2: Laser microscope setup, samples' preparation, and automated milling algorithms

Sample preparation

The DNA-AuNPs origami solution in the buffer was loaded into a glass capillary (61 × 1 × 0.05 mm^3). Both ends of the capillary were sealed with paraffin wax and then centrifuged at 500 rpm for 2 minutes. The centrifugation step was performed to concentrate the DNA-AuNPs material in one end of the capillary. All samples were stored in the refrigerator when not used for experiments. The sample was placed on the Linkam heat stage (LTS420) and thermostated at 45 °C. A good thermal contact between the plate and capillary was facilitated by a small amount of microscope immersion oil. The base temperature was maintained throughout the experiment.

Photothermal processing setup

The photothermal experiments were conducted using a modified optical microscope setup, as described in the previous work.[3] A fiber-coupled (120 µm, multimode) blue laser source (15 W, 450 nm, Opt Lasers) equipped with an achromatic doublet collimator was used to project the beam onto an air-cooled 1024 × 768 digital micromirror device (DMD, 0.45" chip, Texas Instruments). The optical configuration of the microscope with a tube lens focal length f = 200 mm allows projection of the demagnified DMD image onto the sample plane and controlled illumination of selected areas within the microscope FOV. (Figure S5). The optical flux density delivered to the sample is dependent on the effective focal length of the microscope objective and regulated by the duty cycle of the laser modulation. In this study, a typical flux used for AuNP-DNA crystals melting using a 50× objective (Nikon CF Plan) ranged from ≈ 0.05 to 0.26 mW/μm^2 for 10% and 50% laser duty cycle, respectively. Simultaneous microscopic observation of crystals and laser processing is achieved through a long-pass dichroic mirror (cut-on edge at 490 nm) in the laser microscope's illumination path (Figure S5). The mirror's rejection of blue light leads to the characteristic yellow hue seen in the background of the optical micrographs (Figure S6a-c).

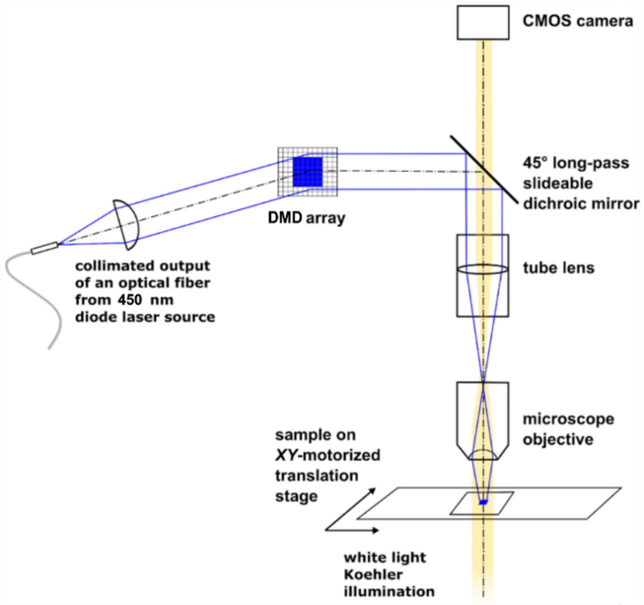

Figure S5. *Schematic of the optical system. Laser illumination rays are shown in blue and white light illumination and imaging rays are shown in yellow.*

Laser milling and drilling experiments

The laser milling experiments were performed by displaying a circular spot (φ = 9–15 µm) in such a way that only the edge of the crystal was illuminated. This approach resulted in the subsequent melting of the illuminated crystal parts, allowing reshaping. Laser drilling was performed by displaying a laser spot of the selected size 1.4, 3.3, 5.8, 9.1 µm diameter with defined power (in the range 0.1–0.25 mW/µm^2) for a specified amount of time (typically 2–5 seconds). This could be achieved either by manually targeting the crystal within the GUI software controlling the microscope or by using an automated method.

To improve the efficiency and precision of the drilling process, we developed an algorithm that detects and guides the re-melting procedure. First, the camera image is analyzed using adaptive thresholding techniques (implemented in OpenCV) to distinguish the crystals from the background (Figure S6a). Once the crystals are isolated, a minimal-area rectangle is fitted around each one to determine its orientation within the field of view (FOV). For cubic-shaped crystals, this rectangle typically coincides with the crystal's shape. For milling, the user provides a desired shape as a PNG file (white shape on a black background), which is then loaded into the program. The algorithm projects this shape onto the selected crystal, determines the optimal rotation angle, and calculates where to place illumination spots around the crystal (Figure S6b). In the example shown, the shape was rectangular and required a rotation of approximately 45° to match the crystal's orientation. For laser drilling, a separate PNG file containing the desired etching (again, white on black) is loaded. This pattern is converted into an array of small circular "illumination spots", which collectively form the final drilling design (Figure S6c). The locations of these spots are then recalculated according to the crystal's orientation by mapping their pattern-based coordinates to absolute positions within the microscope's FOV. Finally, the laser is directed to illuminate each spot with predefined power and duration settings. This method allows the user to select precisely which crystal to mill or drill, thereby enhancing both the precision and control of the re-melting process.

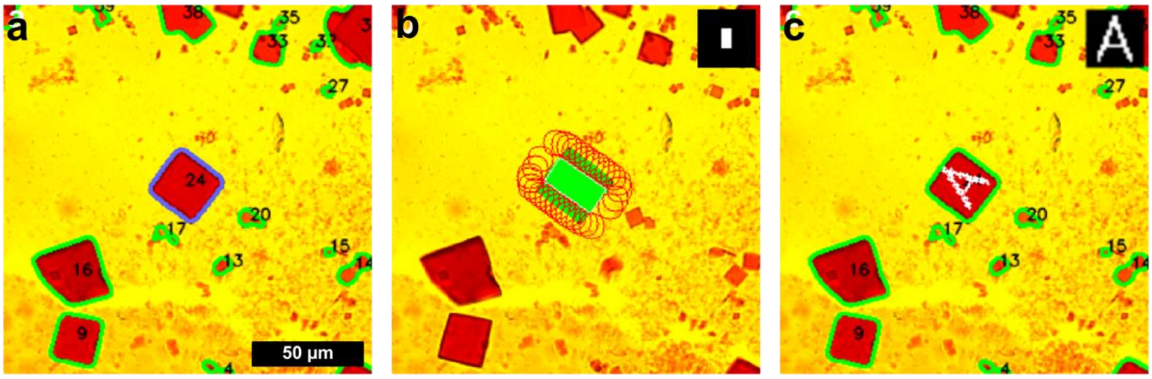

Figure S6. *Automated laser milling and drilling process demonstrating how areas for milling are designated. (a) Microscope image of crystals enumerated by the crystal detection algorithm. (b) Close-up of crystal no. 24 (green overlay) with red circles indicating where the laser will illuminate to transform the crystal into the desired shape. (c) Overlay of illumination spots used during drilling to recreate the letter "A" within the crystal. The insets in (b) and (c) show the patterns as loaded into the program's memory.*

Supporting Note 3: Characterization of photothermal effects and numerical simulations

Laser flux density measurements

The time-averaged optical power of the source is controlled by adjustment of the duty cycle of a 2 kHz 0–5 V square wave PWM voltage signal. Optical flux density delivered to the sample, dependent on the microscope objective demagnification and laser duty cycle is presented in Table S5. All experiments presented here were performed using a 50× objective. Power measurements were performed on a defocused beam using a power meter (PM130D, Thorlabs) equipped with a photodiode sensor (S130C, Thorlabs).

Table S5. *Laser flux density at several laser duty cycles.*

DMD demagnification factor	Laser duty cycle			
	10%	15%	20%	50%
	Optical flux density [mW/µm^2]			
**50 ×**	0.0525	0.0790	0.105	0.263

Numerical simulations of laser heating

To determine the optimal laser power for the laser drilling experiments, we conducted finite-element numerical simulations of the thermal fields induced in AuNP-DNA supercrystals during illumination by light patterns using the Comsol Multiphysics software package. The model crystal used for simulations was a cube with dimensions of 30 × 30 × 30 µm^3. Due to the partial penetration of the crystals by light, a 3D heat transfer model was constructed in which the intensity of light incident on the crystal follows an exponential decay along the optical axis, $A = A_o\, exp(-\alpha z)$, where A_o is the optical flux density at the top surface of the crystal and α is the absorption depth for 450 nm radiation inferred from the spectroscopic measurements. A series of analyses were performed investigating steady-state and time-dependent temperature profiles induced inside the crystal after illumination with various optical flux densities and different laser spot sizes.

To estimate the minimum laser power density needed to melt DNA origami in the presence of absorbing AuNPs, we performed finite element simulations using COMSOL Multiphysics. The simulated configuration was designed to closely match the experimental setup, consisting of a 75 mm × 25 mm × 1 mm glass slide and a thin, rectangular glass capillary (61 mm × 1 mm × 0.05 mm) filled with water. Figure S7a provides a schematic of the experimental setup used in the simulations. The surface-deposited optical power density matched the experimentally measured laser flux density (Table S5), as well as the measured optical absorption of AuNP-DNA crystals described in the main text. Inside this capillary, a 30 µm × 30 µm × 30 µm AuNP-DNA crystal was modeled. A constant temperature boundary condition (T_{base}) was applied to the back surface of the capillary, while the remaining surfaces were subject to a Newtonian convective cooling boundary condition, with a convective heat transfer coefficient $h = 20\ W \cdot m^{-2} \cdot K^{-1}$ and an ambient temperature $T_{amb} = T_{base}$. Although the overall setup included a glass capillary, the simulations themselves focused on the water and DNA regions only, neglecting the thermal contribution of glass. The thermal properties of the modeled materials are listed in Table S6, with the thermal conductivity of glass assumed to be constant over the relevant temperature range. Radiative heat losses were neglected.[3] A tetrahedral mesh was used throughout, with a higher mesh density near the laser-heated region (where the smallest element size was under 1 µm) and a coarser mesh in less critical areas. Both steady-state and time-dependent simulations were carried out. The illuminated zone was a 5.8 µm diameter circle. The optical power

flux density was 0.2 mW/μm^2. Figure S9 illustrates the time-dependent laser heating results. Unless otherwise specified, simulation outputs are reported as a temperature rise, ΔT, above the supporting glass slide temperature, $T_{base.}$ (Figure S7b).

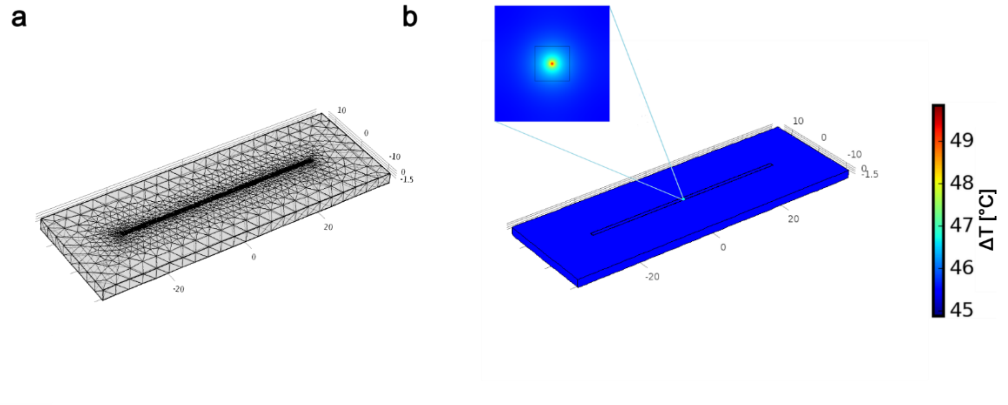

Figure S7. *Finite element simulation of heat transport in the crystal drilling experiment. (a) A meshed model of the experiment sample on a supporting microscope glass. (b) An exemplary result of the simulation – a steady-state temperature rise (ΔT) induced in the sample by illuminating with a single dot shaped 450 nm laser beam.* T_{base} *= 45 °C.*

Table S6*. The thermal properties of the modeled materials.*

Material	Heat capacity, Cp [J/(kg·K)]	Density, ρ [kg/m^3]	Thermal conductivity, k [W/(m·K)]	Absorption coefficient, α [cm^{-1}]
**Water**	4184	1000	0.6	negligible
**Fully-filled AuNP-DNA crystal in water**	129	1100	1.0	435
**Half-filled AuNP-DNA crystal in water**	129	1100	1.0	299

Temperature distribution along the optical axis of the beam

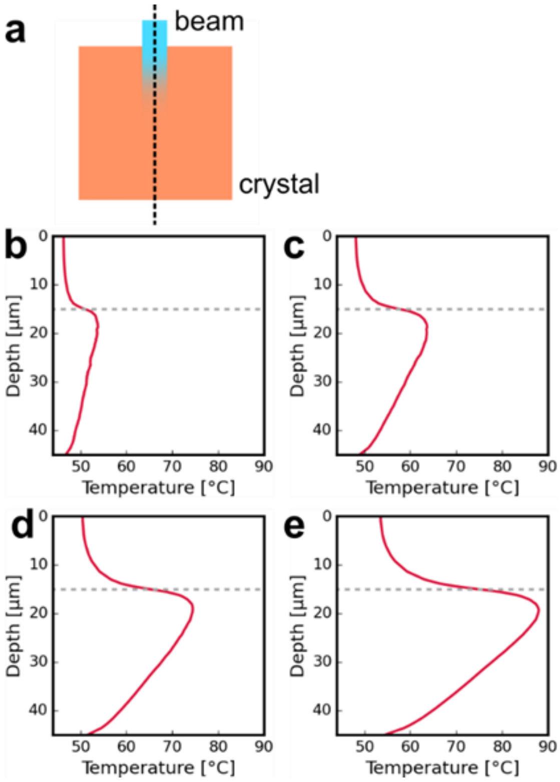

Figure S8. *Steady-state thermal profiles along the z-axis positioned at the center of the beam (through crystal depth) under illumination by 1.4, 3.3, 5.8, and 9.1 μm beam (panels b, c, d, and e respectively). Horizontal dashed lines mark the location of the top surface of the crystal at z = 15 μm. The optical power flux density was 0.2 mW/μm^2.*

Time-dependent photothermal heating simulations

We performed time-dependent simulations to estimate the characteristic time τ for the crystal to reach a steady-state temperature, assuming it remains in the condensed phase even above the lattice melting temperature. As shown in Figure S9, τ is on the order of 100 ms. In contrast, the experimentally observed time to drill a hole in the illuminated crystal is approximately 5 s. Therefore, the process is not limited by heat transfer but rather by the slower kinetics of lattice melting, which ultimately governs how quickly AuNP-DNA crystals can be processed with laser beams.

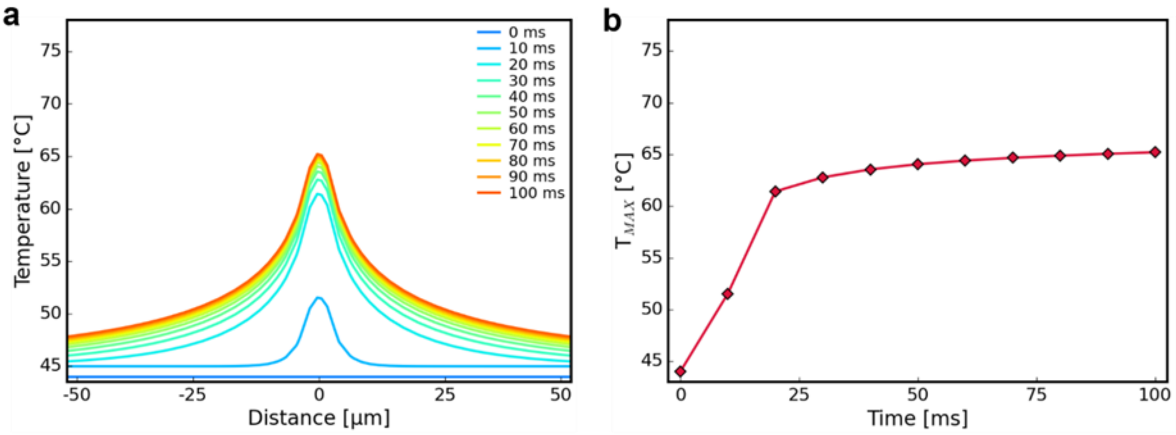

Figure S9. *Numerical simulation of time-dependent laser heating of the 30 × 30 × 30 μm^3 AuNP-DNA crystal. (a) Temperature profiles at different times after the start of illumination. (b) Temperature increase at the top surface of the illuminated crystal over time. The illuminated zone was a 5.8 μm diameter circle. The optical power flux density was 0.2 $mW/\mu m^2$. The temperature reaches 99% of the plateau value within ~100 ms from the start of illumination.*

The effect of absorbing AuNPs-containing frames removal

The laser drilling process is self-limiting. As the light-absorbing AuNPs-DNA lattice melts and AuNPs rapidly diffuse away from the hole, the overall process temperature drops, eventually falling below the lattice melting point. Figure S10 presents the results of a numerical simulation of the temperature in the crystal after removing 25%, 50%, and 75% of the light-absorbing material from the top of the crystal core.

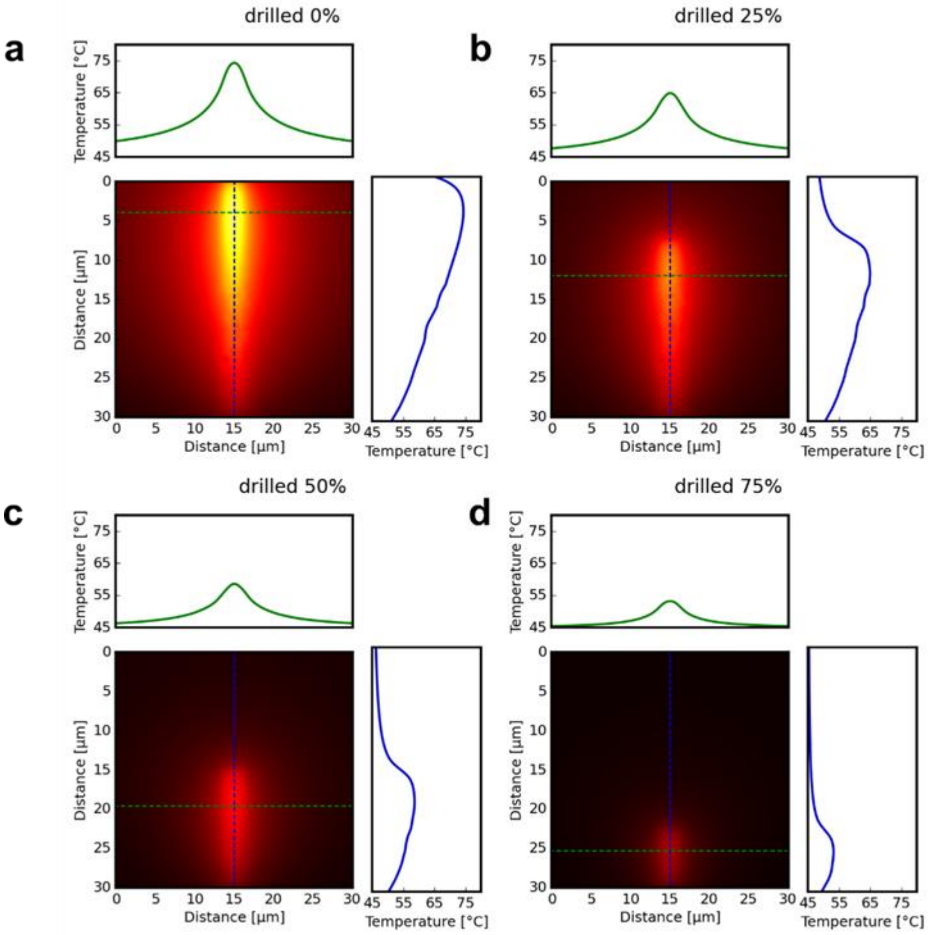

Figure S10. *The effect of gold nanoparticle removal from the core of laser-drilled hole. Simulated 2D thermal fields and 1D temperature cross-sectional profiles under illumination by a 5.8 µm beam spot size at 0.2 mW/µm*^{2} *at the beginning of illumination (a), after removing 25%, 50%, and 75% of light-absorbing material from the top of the crystal core (b–c). The diameter of the hole forming in the crystal was determined to be 5.6 µm (see Fig. 2e).*

Half-filled AuNP-DNA crystals

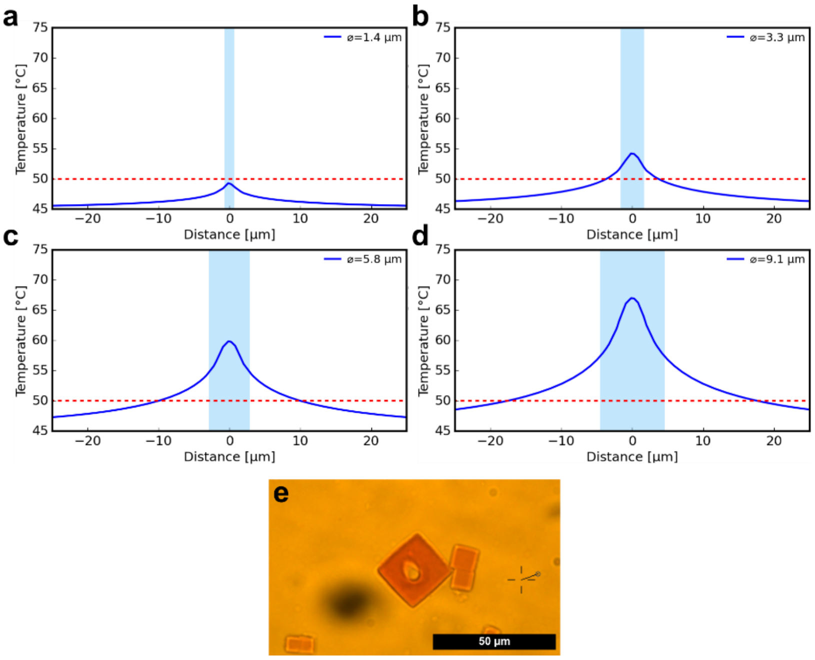

Figure S11. *Laser drilling in DNA-origami crystals half-filled with AuNPs. (a-d) Simulated temperature profiles at the top surface of the crystal illuminated with beams with flux intensity of 0.2 mW/µm*^{2} *and the diameter of 1.5, 3.3, 5.8, and 9.1 µm. The blue region line indicates the diameter of the laser beam for each simulation. (e) The result of the experiment in which a 3.3 µm beam with flux intensity (0.25 mW/µm*^{2}*) was used to drill a hole in a crystal.*

Simultaneous drilling in two spots

Simultaneous illumination of two spots produces two distinct holes, provided the beams are spaced far enough apart. Figure S12 shows both the experimental results and the corresponding finite element simulations of two-spot laser processing. The experiments were conducted using a laser beam with a diameter of 3.3 μm and an optical power flux density of 0.2 mW/μm², while varying the center-to-center spacing d from 0 to 20 μm under constant illumination. Because the fluxes of two overlapping spots do not add, the minimum temperature is observed at $s = 0$. For two-spot drilling, the smallest spacing required to produce two separate circular holes is approximately 10 μm. When the spacing is below this threshold, the holes merge, resulting in the figure-eight-shaped hole shown in Figure S12d.

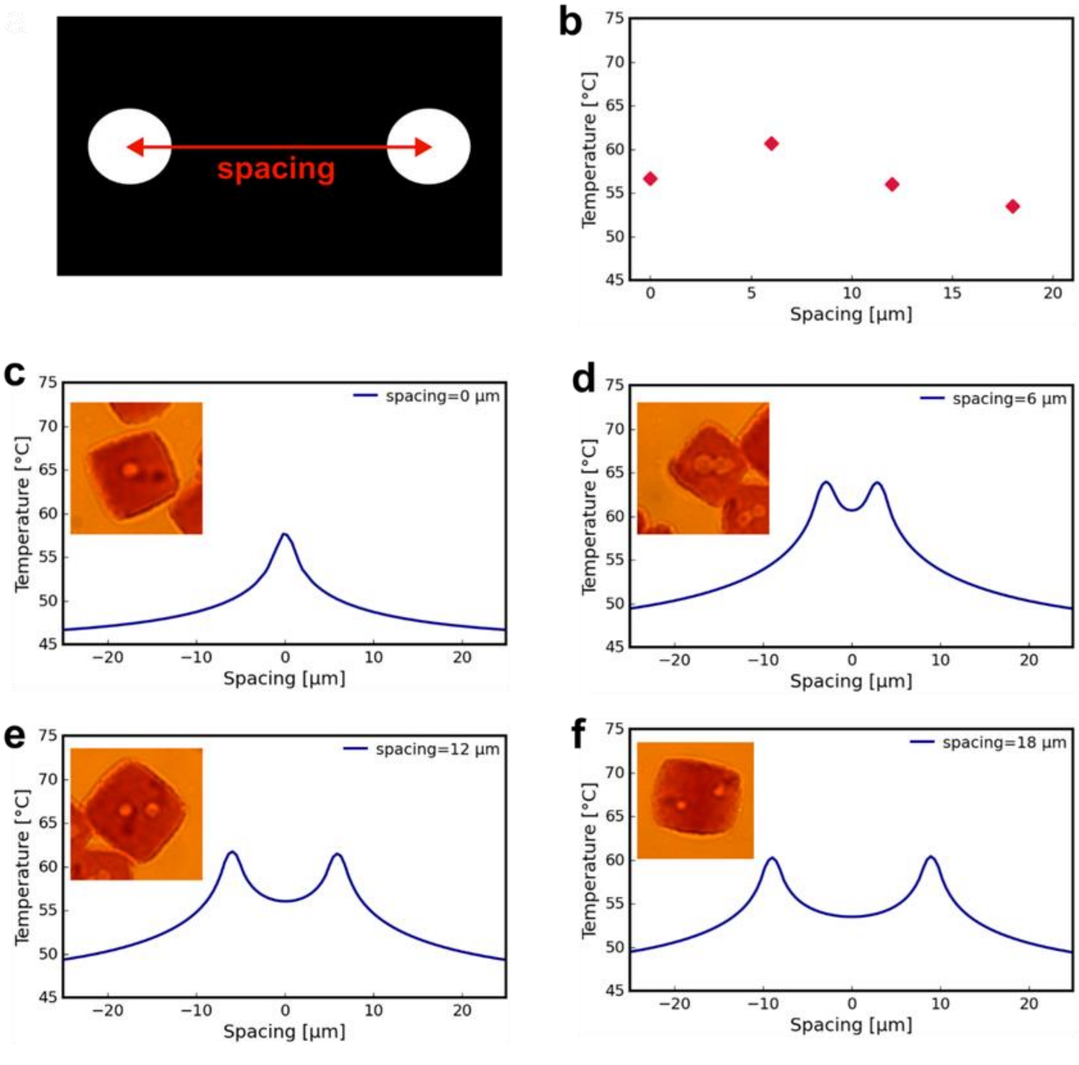

Figure S12. *Simultaneous drilling with the two-spot pattern. (a) Graphical representation of the two-spot pattern used in the experiment. (b) The temperature at the top surface at the center of the crystal between the two spots in relation to their spacing (center-to-center distance). Panels c – f show the results of the laser drilling experiment and simulated surface temperature profiles obtained for various distances between the spots. The flux over the illuminated area was constant (0.2 mW/μm²), hence the drop in temperature for s = 0 (single spot illumination). The diameter of each spot was 3.3 μm.*

Supporting Note 4: Confocal imaging and image post-processing routines

Confocal fluorescence microscopy

Imaging and analysis of the fine structure of AuNP-DNA crystals after laser sculpting were conducted with a Nikon A1R MP confocal microscope (Nikon Corporation, Tokyo, Japan) equipped with Plan Apo VC 60×/NA1.4 objective. The use of fluorescent dyes was not necessarily due to the autofluorescence of the crystals observed after excitation with a 488 nm laser. Images were acquired at 2040 × 2048 pixels resolution using a *z*-step of 0.21 μm. The green autofluorescence was recorded with a green bandpass filter (500–550 nm). For the image analysis and processing of the 2D and 3D images, Nikon NIS Elements 4.10 software was used. The reconstructed 3D images of Au-DNA crystals were rendered after stacking the collected confocal images.

The post-processing procedure was realized in NIKON NIS Elements 4.10 software and consisted of the following steps:
1) Image/Smooth for denoising,
2) Histogram/LUTs edition,
3) 3D Volume View or View/Image/Slices View/Maximum Intensity Projection.
Alternatively, ImageJ (Fiji) image processing software was used to render the final images utilizing Open Hyperstack, Image/Stacks/Z Project, and Max Intensity or Sum Slices protocols.[4]

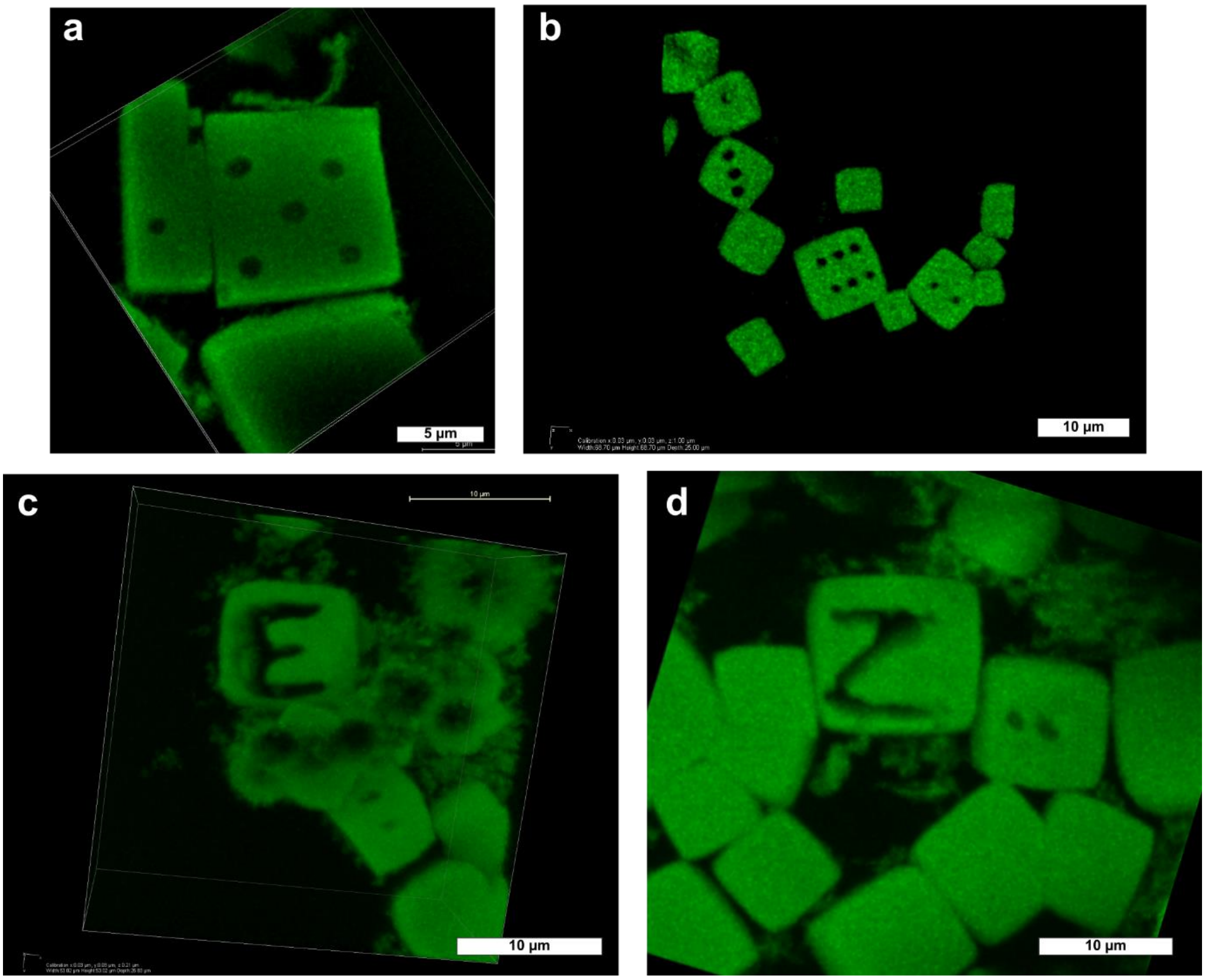

Figure S13. *Confocal fluorescence images of AuNP-DNA crystals collected using 488 nm excitation wavelength and a green bandpass filter (500 – 550 nm).*

Supporting Note 5: Superlattice silica coating and scanning electron microscopy

Superlattice silica coating

Laser-drilled DNA origami superlattices were extracted from a glass capillary and placed on a glow-discharged silicon substrate. Separately, a silica coating reaction mixture was prepared by mixing a 0.1×TAE buffer with 10 mM $MgCl_2$, 2 μl of (3-aminopropyl)-triethoxysilane and 7 μl of tetraethoxysilane into a final volume of 1 ml. After letting the superlattices settle for 5 minutes, the substrate was submerged in a PCR tube containing 225 μl of silica coating mixture and placed on a thermoshaker at 700 rpm for 4 hours. The substrate was then removed, washed with DI water, and air-dried.

FIB-SEM imaging

SEM images were taken using a FEI Helios Nanolab 660 FIB-SEM. Silica-coated superlattices on a silicon substrate were mounted onto the stage for imaging. Individual crystals with holes were located and imaged. To perform cross-sectional imaging experiments, superlattices were coated with a 1–2 μm layer of platinum and then cut with 15 nm slices. Cross-sectional imaging was performed at a 52° angle. For 3D cross-sectional image stacks, image registration and processing were performed using the MultiStackReg plugin in ImageJ software.[4]

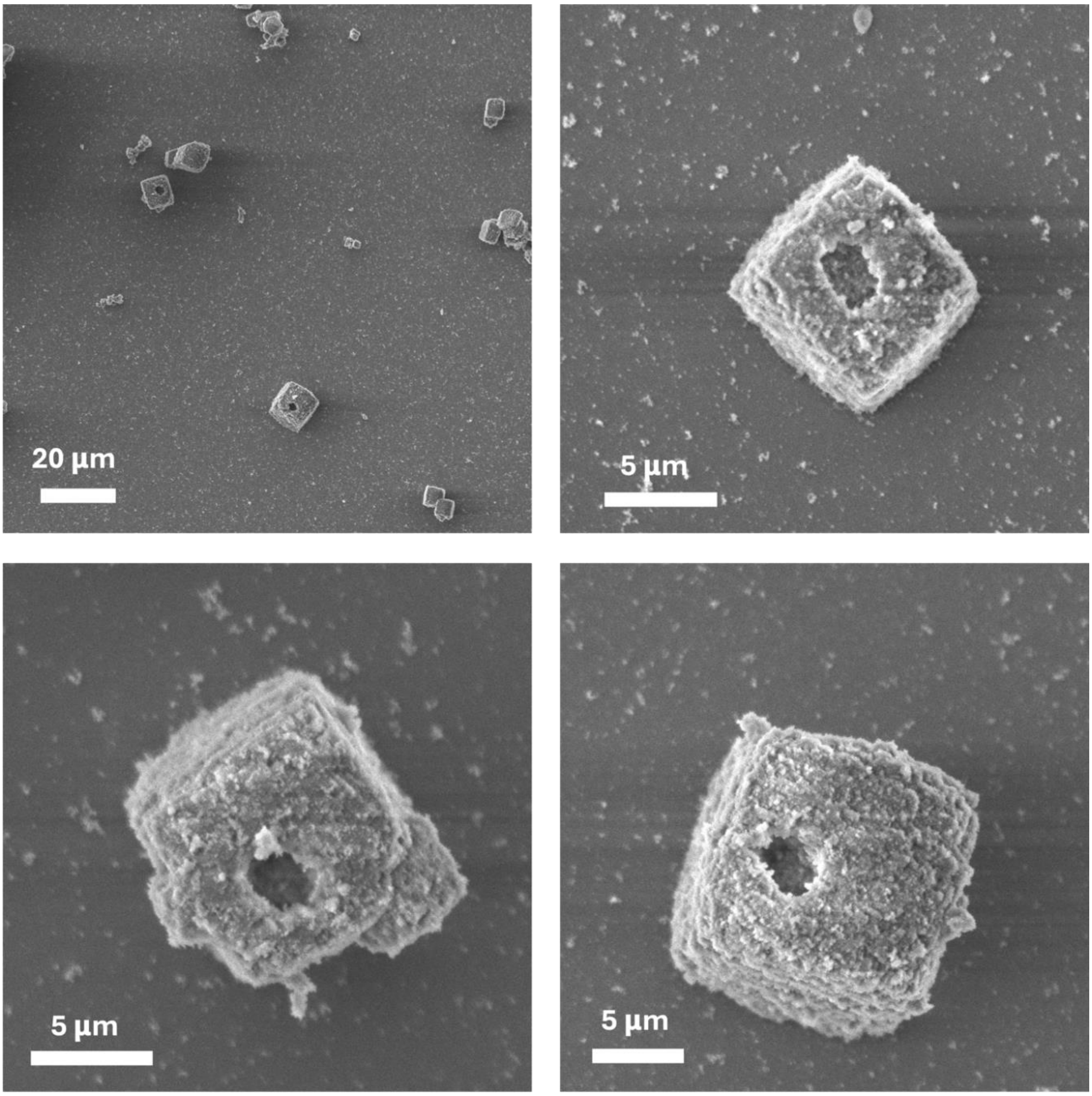

Figure S14. *Representative SEM images of laser-drilled DNA-assembled crystals after silica coating.*

Description of supplementary video files

1. SI Movie S1 – movie collected during manually controlled light-drilling of single holes in the AuNPs-DNA crystals

2. SI Movie S2 – movie collected during automated light drilling of multiple holes in the AuNPs-DNA crystals

3. SI Movie S3 – movie collected during automated side-milling for shaping the AuNPs-DNA crystals

4. SI Movie S4 – FIB-SEM tomographic reconstruction showing 3D crystal structure frame-by-frame.

List of references

1. Tian, Y. *et al.* Ordered three-dimensional nanomaterials using DNA-prescribed and valence-controlled material voxels. *Nat. Mater.* **19**, 789–796 (2020).
2. Yager, K. G., Zhang, Y., Lu, F. & Gang, O. Periodic lattices of arbitrary nano-objects: modeling and applications for self-assembled systems. *J. Appl. Crystallogr.* **47**, 118–129 (2014). https://github.com/CFN-softbio/ScatterSim
3. Szustakiewicz, P., Powała, F., Szepke, D., Lewandowski, W. & Majewski, P. W. Unrestricted Chiral Patterning by Laser Writing in Liquid Crystalline and Plasmonic Nanocomposite Thin Films. *Adv. Mater.* **36**, 2310197 (2024).
4. Schindelin, J. *et al.* Fiji: an open-source platform for biological-image analysis. *Nat. Methods* **9**, 676–682 (2012).
